# Analytical Strategies for Green Extraction, Characterization, and Bioactive Evaluation of Polyphenols, Tocopherols, Carotenoids, and Fatty Acids in Agri-Food Bio-Residues

**DOI:** 10.3390/molecules30061326

**Published:** 2025-03-15

**Authors:** David Vicente-Zurdo, Esther Gómez-Mejía, Sonia Morante-Zarcero, Noelia Rosales-Conrado, Isabel Sierra

**Affiliations:** 1 Departamento de Tecnología Química y Ambiental, Escuela Superior de Ciencias Experimentales y Tecnología (E.S.C.E.T), Universidad Rey Juan Carlos, C/Tulipán s/n, 28933 Móstoles, Spain; david.vicente.zurdo@urjc.es (D.V.-Z.); sonia.morante@urjc.es (S.M.-Z.); 2 Departamento de Química Analítica, Facultad de Ciencias Químicas, Universidad Complutense de Madrid, Av Complutense s/n, 28040 Madrid, Spain; egomez03@ucm.es; 3 Instituto de Investigación de Tecnologías para la Sostenibilidad, Universidad Rey Juan Carlos, C/Tulipán s/n, 28933 Móstoles, Spain

**Keywords:** analytical chemistry, green extraction, characterization, bioactive compounds, polyphenols, tocopherols, carotenoids, fatty acids, agri-food bio-residues, circular economy

## Abstract

Recent advancements in analytical strategies have enabled the efficient extraction and characterization of bioactive compounds from agri-food bio-residues, emphasizing green chemistry and circular economy principles. This review highlights the valorization of several agri-food bio-residues for the extraction of high-value-added bioactive compounds, particularly polyphenols, tocopherols, carotenoids, and fatty acids, as a biorefinery approach. To this end, the adoption of environmentally friendly extraction technologies is essential to improve performance, reduce energy consumption, and minimize costs. This study therefore examines emerging methodologies such as supercritical fluid extraction, pressurized liquid extraction, pulsed electric fields, and matrix solid-phase dispersion, highlighting their advantages and limitations. Additionally, the chemical characterization of these bioactive compounds is explored through spectrophotometric and high-resolution chromatographic techniques, crucial for their accurate identification and quantification. This is complemented by an analysis of bioactivity assays evaluating antioxidant, antimicrobial, anticancer, neuroprotective, and anti-inflammatory properties, with a focus on their applications in the food, pharmaceutical, and cosmetic industries. However, the analytical control of toxic compounds, such as alkaloids, in these bio-residues is undoubtedly needed. Ultimately, this approach not only promotes sustainability but also contributes to the development of eco-friendly solutions in various industries.

## 1. Introduction

The circular economy and the achievement of Sustainable Development Goals (SDGs) are increasingly intertwined, particularly in the context of valorizing agri-food bio-residues [1]. These residues, often considered waste, are now recognized as valuable sources of bioactive compounds such as polyphenols, tocopherols, carotenoids, carbohydrates, proteins, bioactive peptides, lignin, and fatty acids, among others [2], which exhibit a range of bioactive properties, including antioxidant, antibacterial, antifungal, anticancer, neuroprotective, and anti-inflammatory activities [3]. These compounds exhibit strong antioxidant activities, which help neutralize free radicals and reduce oxidative stress, a key factor in the development of chronic diseases [4]. Their antibacterial and antifungal properties make them effective in inhibiting the growth of harmful microorganisms, thus enhancing food safety and shelf life [5]. Additionally, their anticancer properties are attributed to their ability to modulate various cellular pathways, inducing apoptosis and inhibiting tumor growth [6]. Neuroprotective effects are also notable, as these compounds can protect neurons from oxidative damage and inflammation, potentially reducing the risk of neurodegenerative diseases [2,7]. Furthermore, their anti-inflammatory properties help in mitigating inflammation, which is a common underlying factor in many chronic conditions [5]. The valorization of agri-food bio-residues using a biorefinery approach not only aligns with the principles of the circular economy but also contributes to the fulfillment of several SDGs by promoting sustainable practices and reducing environmental impact.

The extraction of bioactive compounds from agri-food bio-residues has significant applications across various industries, including food, pharmaceutical, and cosmetic sectors. Extracts rich in polyphenols, tocopherols, carotenoids, and fatty acids are particularly valuable due to their diverse health benefits and functional properties. However, the efficient and sustainable extraction of these compounds presents several challenges. The inherent complexity and heterogeneity of these matrices, coupled with the presence of undesirable interfering substances and the potential degradation of target compounds under processing conditions, necessitate the development of refined extraction strategies. Additionally, minimizing solvent consumption while maintaining an optimal cost–benefit balance remains a key technical constraint [8,9]. As such, striking a synergy between efficiency, sustainability, and economic viability is a persistent challenge, driving continuous advancements in extraction methodologies to enhance the valorization of these underutilized resources. In response to these challenges, green extraction methods such as ultrasound-assisted extraction (UAE) [10], microwave-assisted extraction (MAE) [11], supercritical fluid extraction (SFE) [12], pressurized liquid extraction (PLE) [13], pulsed electric fields (PEF) [14], and matrix solid-phase dispersion (MSPD) [15], as well as hybrid and sequential extraction approaches, are increasingly being explored to enhance the yield of bioactive compounds while minimizing environmental impact [8]. These methods are currently being optimized or developed at both laboratory and industrial scales, where both theoretical efficiency and practical feasibility are paramount for large-scale applications.

The characterization of these bioactive compounds requires advanced analytical techniques. Separation methods such as liquid chromatography (LC) and gas chromatography (GC), coupled with detection techniques like mass spectrometry (MS), diode array detectors (DADs), and fluorescence, are essential for identifying and quantifying individual compounds [16]. Despite advancements, challenges remain in lowering detection and quantification limits, broadening the screening of multiple analytes simultaneously, and reporting detailed phenolic profiles rather than just total polyphenol content (TPC) [17]. These detailed profiles are crucial as the bioactive properties can vary significantly among different phenolic compounds.

Evaluating the bioactive properties of these extracts involves various in vitro methods [18]. Standardizing these evaluation methods and reporting results in a comparable manner across different studies is essential for advancing the field and ensuring the reliability of the findings [19]. Addressing these challenges will pave the way for more effective utilization of agri-food bio-residues, contributing to the development of innovative, health-promoting products and supporting the broader goals of sustainability and a circular economy.

**Figure 1:**
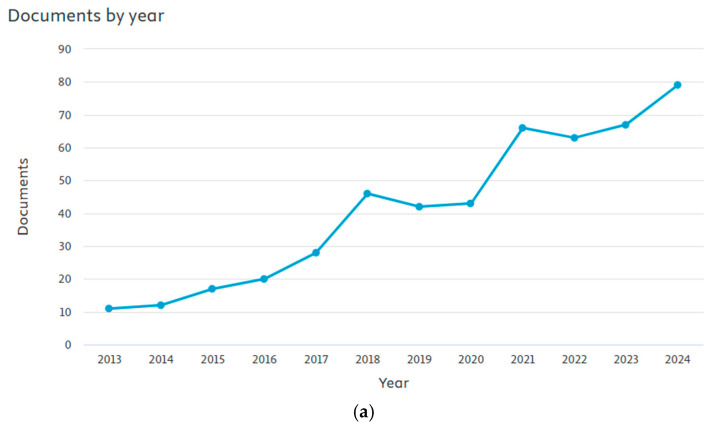

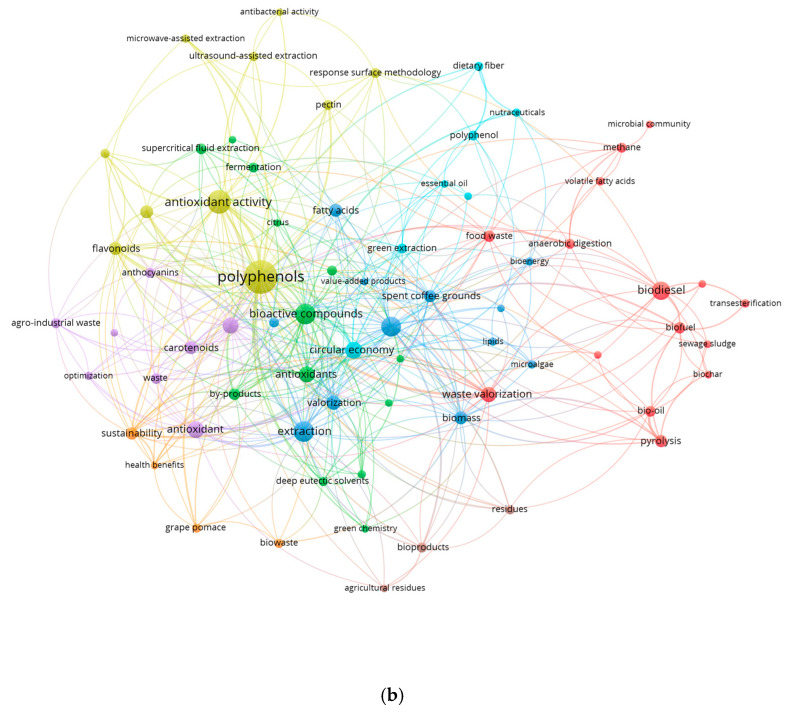
Documents by year found in Scopus using the sequence of keywords described in the methodology (**a**). VOSviewer software version 1.6.20 plot showing the co-occurrence of keywords in the 618 documents found in Scopus related to this study (2013–2024) (**b**).

Although the beneficial effects of these families of natural molecules have been discussed separately in the literature, it is imperative to conduct a comprehensive interpretation when these molecules coexist within the same agri-food bio residue. This necessitates an intensive review of extraction methods that maximize the quantities obtained, techniques that enable the simultaneous characterization of the analytes of interest, and the evaluation of various bioactive properties of the extracts due to the wide range of bioactives they exhibit. This review provides a recent holistic overview of the current state of green extraction, characterization, and evaluation of the bioactive properties of agri-food bio-residues rich in polyphenols, tocopherols, carotenoids, and fatty acids. The selection of studies was mainly conducted using Scopus and ScienceDirect as reference databases. The exploration approach applied the following sequence of keywords: (Extraction or Characterization or Bioactivity), (Polyphenols or Tocopherols or Carotenoids or (Fatty and Acids)), and Waste and Residues. Inclusion criteria were limited to articles published in English. Duplicates were deleted to exclude any redundancy. Additionally, studies deemed to offer substantial contributions to the field were included for thorough analysis. The graph of the documents per year, reported from Scopus, demonstrates the growth of articles in this area since 2013 (Figure 1a). Furthermore, Figure 1b shows the co-occurrence of keywords in these articles, where their interactions with “Polyphenols”, “Antioxidant activity”, “Extraction”, and “Circular economy” stand out. This network visualization underscores the interconnectedness and thematic clustering of these key concepts, suggesting a robust interdisciplinary focus on the extraction processes and antioxidant properties of polyphenols within the framework of sustainable and circular economic practices.

## 2. Bioactive Compounds Classification

Over the past decade, research on bioactive compounds has surged, underscoring their pivotal role in human health. Scientific evidence has increasingly illuminated the potential of bioactive compounds, found in foods and plants, to name but a few, to prevent chronic diseases and enhance overall well-being.

The question, therefore, arises: what is a bioactive compound? A bioactive compound is an intrinsic physiologically active substance found in consumables or other non-food sources, including meat, dairy, mycological organisms, fruits, vegetables, and plant-based goods, as well as their waste. These naturally occurring molecules, some of them lacking nutritional value, have the capacity to engage with various components of living tissue, thereby exhibiting a range of potential health benefits. Additionally, they are predominantly produced by plants as secondary metabolites and are utilized in various functions such as competition, defense, attraction, and signaling [20,21].

Further, the term bioactive compound encompasses a magnificent array of chemicals valuable for the development of new medicines, nutraceuticals, food ingredients, and food packaging, meeting the growing consumer interest in health promotion and natural, eco-friendly food products. Within this pool are polyphenols, tocopherols, carotenoids, fatty acids, terpenes, proteins, xanthine alkaloids, saponins, and some others [21,22].

### 2.1. Polyphenols

Polyphenols, also referred to as phenolic compounds, constitute a varied group of naturally occurring substances distinguished by the presence of multiple phenol units. Their consumption through diet is associated with numerous health benefits [23]. These bioactives represent one of the most intriguing, prevalent, and widely distributed groups of phytochemicals in the plant kingdom, as well as in the fungal domain [20,21]. Phenolic compounds form a class of secondary metabolites synthesized primarily by plants, as well as algae and fungi, serving as a multifaceted tool for defense and signaling against various abiotic and biotic stressors. Additionally, they contribute to the intrinsic coloration of vegetation, as well as to the organoleptic profiles, flavors, odors, and bitterness of fruits and vegetables [24].

**Figure 2:**
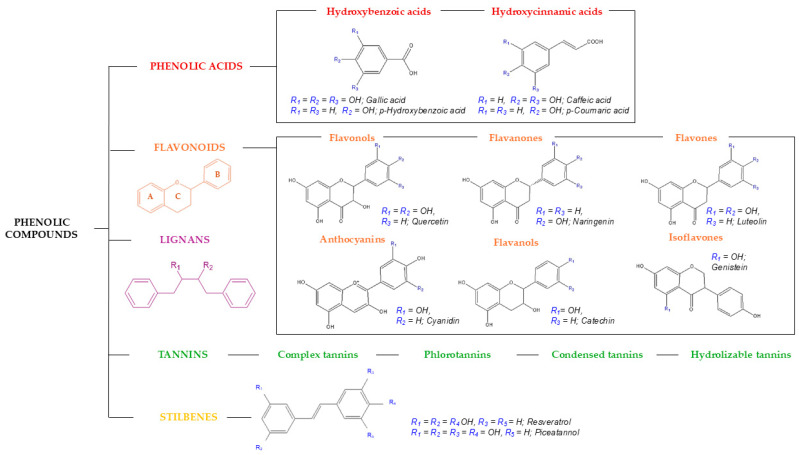
Classification and structure of principal phenolic families: phenolic acids (red color), flavonoids (orange color), lignans (purple color), tannins (green color) and stilbenes (yellow color).

Phenolic compounds are the subject of extensive research due to their high prevalence in natural sources and their diverse bioactives, which have garnered the attention of nutritionists, researchers, and various industrial sectors. Polyphenols exhibit multiple bioactives, with their potent antioxidant property standing out, attributed to their ability to regulate the generation of reactive oxygen species and reactive nitrogen species [24,25]. Consequently, phenolic compounds effectively neutralize the aforementioned reactive species by acting as hydrogen donors and reducing agents. Also, they are capable of chelating metals, which stops oxidizing species from forming [25]. Furthermore, polyphenols exhibit anti-inflammatory, antiproliferative, neuroprotective, and antimicrobial properties, among others. Overall, these bioactives have been investigated for their potential application in the prevention and alternative treatments of various diseases, as well as in the development of functional foods and cosmetic ingredients, among other uses [22]. In terms of chemical characteristics, their basic phenolic skeleton can exhibit a vast array of diverse variations, underscoring the heterogeneity and diversity of compounds within this family, with 8000 different phenolic structures reported as early as 2010. To simplify their classification and systematization, a structural criterion can be employed, based on the number of phenolic rings and the structural elements attached to them [21,26]. Consequently, five main groups can be established: phenolic acids, flavonoids, lignans, stilbenes, and tannins, as illustrated in Figure 2.

Phenolic acids represent the simplest polyphenols, with a phenolic structure attached to a carboxylic functional group. They can be further classified into hydroxybenzoic acids (e.g., gallic acid) and hydroxycinnamic acids (e.g., caffeic acid) [16]. These are abundant in vegetables, fruits, and grains, and are commonly found as esters of hydroxy acids, namely quinic, shikimic, and tartaric acids, as well as sugar derivatives [21,26].

Further to this, two phenolic rings (A and C) connected by an oxygenated three-carbon heterocycle (B) define flavonoids, the most extensive family of phenolic compounds (Figure 2). Based on their heterocyclic ring variations and hydroxylation patterns, they are divided into six major subclasses: flavonols, flavones, flavanones, flavan-3-ols, isoflavones, and anthocyanins [24,26]. Owing to their extensive range of bioactives, flavonoids are ubiquitously present in numerous food matrices and are the subject of extensive research [27]. The configuration, substitution patterns, and quantity of hydroxyl groups within these molecules play a pivotal role in determining their antioxidant capacity and overall bioactivity [26]. Flavonols are flavonoids characterized by a double bond between C2 and C3, with a hydroxyl group attached at C3 and a carboxyl group at C4. Many edible and therapeutic plants contain flavonols; two prominent examples are quercetin and kaempferol. Flavones, conversely, possess a double bond between C2 and C3, yet they lack a hydroxyl group at C3, rendering their structure akin to that of flavonols. Luteolin and apigenin are the most prevalent flavones, typically found in vegetables and fruits, particularly citrus varieties. Flavanones are chemically distinguished by the presence of a carboxyl group at position 4, without a double bond between C2 and C3. Citrus fruits and grapes are the main sources of these chemicals, with naringin, naringenin, and hesperidin being the most researched [21,26]. The largest subclass of flavonoids is represented by flavanols, also known as flavan-3-ols. The presence of a functional hydroxyl group at position 3 is the only characteristic that distinguishes this class, where catechin and epicatechin are notable examples. As for the sources of flavanols, tea prevails as one of the richest dietary sources of these compounds. Regarding isoflavonoids, these compounds are distinguished by their benzenoid substituent at position 3, which imparts a structure reminiscent of endogenous estrogens. Predominantly found in leguminous plants, daidzein and genistein are the principal isoflavones present in soy. Lastly, anthocyanins are derived from flavonols, structurally characterized as flavylium ions. These compounds are responsible for the red-purple hues in various fruits, vegetables, and flowers, with cyanidin, delphinidin, and malvidin being the most prevalent compounds [21,26].

Concerning stilbenes, these polyphenols are characterized by two phenolic rings connected by a two-carbon methylene bridge (C6-C2-C6). Stilbenes are present in the human diet only in small quantities, primarily in the form of resveratrol, which has been extensively studied for its bioactive properties [24,28].

Meanwhile, lignans represent a category of natural phytoestrogens composed of two interconnected phenylpropanoid units. These compounds can appear as aglycones and glycosides in seeds and nuts, with secoisolariciresinol and matairesinol being the most prevalent dietary lignans [24,29].

Finally, tannins, which are high-molecular-weight polyphenols, have recently been cataloged into complex tannins, phlorotannins, condensed tannins, and hydrolyzable tannins (gallotannins and ellagitannins). Unlike ellagitannins, which consist of at least two galloyl units linked by C–C bonds without a glycosidically attached catechin unit, gallotannins are composed of galloyl units or their metadespidic derivatives bound to various polyol, catechin, or triterpenoid units. Additionally, condensed tannins, also known as proanthocyanidins, are oligomers formed from a flavanol core. Phlorotannins, which have phloroglucinol as their common structural base, are found in brown algae, and complex tannins show a catechin unit glycosidically linked to an ellagitannin or gallotannin unit [21,30].

### 2.2. Tocopherols

**Figure 3:**
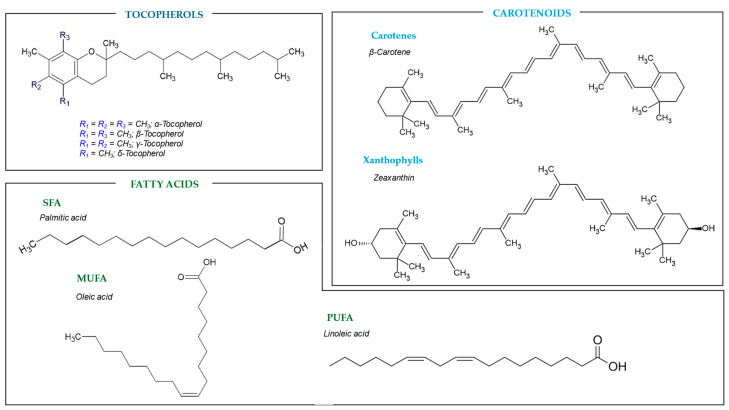
Classification and structure of tocopherols, carotenoids, and fatty acids.

Vitamin E is a group of lipid-soluble compounds that includes both tocopherols and tocotrienols. Specifically, tocopherols are antioxidant compounds with a chromanol ring and a hydrophobic side chain of sixteen carbon, derived from a phenyl group. These phytochemicals play a crucial role in human health by protecting unsaturated fatty acids from lipid peroxidation and ensuring the stability of lipid membranes [31]. Beyond their antioxidant activity, tocopherols are integral to protein–membrane interactions, signal transduction, and gene expression. Furthermore, these phytochemicals have been reported to regulate immune function, facilitate DNA repair, and exhibit potent anti-inflammatory effects [32], which turns them into highly suitable functional ingredients for the pharmaceutical, cosmetic, and nutraceutical industries. Based on the number and position of methyl groups on the chromanol ring, tocopherols are divided into four main types: α-tocopherol, β-tocopherol, γ-tocopherol, and δ-tocopherol (Figure 3).

Tocopherols are localized in the lipid-rich regions of cells, including mitochondrial membranes, fat depots, cholesterol, and low-density lipoproteins. Consequently, the most abundant natural sources of tocopherols are vegetable seeds and oils derived from oilseeds and nuts. While fruits and vegetables do contain tocopherols, their bioavailability is diminished due to their lower lipid content [31,32]. Specifically, α-tocopherol, the most biologically active form, is predominantly found in olive and sunflower oils [33]. α-tocopherol possesses potent antioxidant properties that reduce the risk of cardiovascular diseases and certain types of cancer [34]. β-tocopherol is less common and is found in some vegetable oils. β-tocopherol, while also an antioxidant, is less effective, with only about one-third the efficacy of α-tocopherol in cellular protection [35]. Regarding γ-tocopherol, the most common form in the American diet, it is present in soybean and corn oils and exhibits both antioxidant and anti-inflammatory properties, though its efficacy is approximately 15% of that of α-tocopherol. However, it presents higher cancer-preventive properties than the former [35]. δ-tocopherol, found in lower concentrations in various oils [36], although the least active, still contributes to cellular protection through its antioxidant capabilities. Elevated levels of α-tocopherol may undesirably diminish γ-tocopherol levels, with a higher efficacy in preventing myocardial disease and cancer proliferation. Furthermore, it has been reported that α- and γ-tocopherol exhibit antagonistic effects, with a higher γ–α concentration ratio being associated with an increased risk of obesity [32]. Some authors reported their role in the protection against neurodegenerative diseases such as Alzheimer’s and Parkinson’s diseases [2]. Therefore, it is imperative not only to ascertain the total tocopherol content but also to evaluate the specific content and ratio of each isoform when evaluating a source of these phytochemicals [3].

These findings highlight the complex interplay between different tocopherol homologs and their impact on health, underscoring the need for precise quantification and balance in dietary sources. Understanding the tocopherol content in agri-food bio-residues not only contributes to waste valorization but also promotes the development of functional foods and nutraceuticals.

### 2.3. Carotenoids

Carotenoids represent the most extensive group of naturally occurring lipid-soluble pigments, responsible for the yellow, orange, and red color in many fruits, vegetables, and flowers, being present in every photosynthetic organism, whether bacteria or plants [37]. Chemically, carotenoids consist of eight isoprene units, with cyclic or linear structures at both ends of the carbon chains. As a result, both cis and trans isomers are formed, highlighting the prevalence of the *trans* isomer in nature [38]. Carotene and the xanthophylls are the two primary families of carotenoids that can be identified (Figure 3). Examples of carotenes are β-carotene (a precursor of vitamin A), α-carotene, γ-carotene, and lycopene, featured as hydrocarbons that are highly soluble in organic solvents and insoluble in polar solvents. On the other hand, carotenes’ oxygenated derivatives, known as xanthophylls, dissolve in both organic and polar solvents. Lutein, zeaxanthin, and violaxanthin are a few of the most well-known xanthophylls [38,39].

These compounds are essential for preventing the organism or plant from being photosensitized by its own chlorophyll, in addition to serving as auxiliary pigments in photosynthesis. Further to this, the provitamin A activity of carotenoids, which categorizes them as natural antioxidants, and their contribution to the prevention of cancer, cardiovascular disease, macular degeneration, and chronic degenerative diseases, as well as the development of cataracts, have boosted their application as nutritional supplements and natural colorants in the agri-food and nutraceutical sectors. Be that as it may, these bioactives’ poor water solubility, low bioavailability, chemical instability, and high oxidation sensitivity limit their application [39].

### 2.4. Fatty Acids

Fatty acids are aliphatic monocarboxylic acids that serve as the building blocks of lipids. Based on the number of double bonds, these fatty acids are categorized as saturated (SFA; e.g., palmitic acid and stearic acid), monounsaturated (MUFA; e.g., oleic acid), or polyunsaturated (PUFA) (Figure 3). Their carbon chains vary in length, spanning from four to twenty-eight carbons. While fatty acids can exist in their free form, they are predominantly found in significant quantities within seeds and seed oils, where they are typically bonded into more complex compounds through ester or amide linkages, mainly as triglycerides [40,41].

PUFAs, characterized by having two or more double bonds, are classified as long-chain fatty acids due to their aliphatic tails of sixteen or more carbons. The two principal families of PUFAs are omega-3 (n-3) and omega-6 (n-6). Among these, α-linolenic acid, from the omega-3 family, and linoleic acid, from the omega-6 family, (Figure 3) are essential for human health and must be obtained through dietary sources. The main n-6 family chemical, linoleic acid (C18:2), is widely distributed in nature and may be found in most plant seeds. It is also highly present in oils such as corn and sunflower oil. On the other hand, the primary omega-3 family molecule, α-linolenic acid (C18:3), is less prevalent and mostly found in flaxseed, rapeseed, and soybean oils [41]. These fatty acids play a pivotal role in the prevention of cardiovascular diseases by lowering the concentration of low-density lipoproteins in blood vessels, thereby reducing arrhythmia, mortality from coronary heart diseases, atherosclerosis, and blood pressure. Further to this, PUFA significantly enhances healthy lipid profiles in oils, contributing to metrics such as the desirable fatty acid index, the hypocholesterolemic–hypercholesterolemic ratio, and the indices of atherogenicity and thrombogenicity [3].

Other beneficial long-chain fatty acids include conjugated linoleic acid and pinolenic acid, which are mainly *trans* positional and spatial isomers of conjugated linoleic acid. Although *trans* fatty acids are generally considered detrimental to health, conjugated linoleic acid has garnered attention for its potential beneficial effects in mitigating chronic diseases [42].

## 3. Sources of Bioactive Compounds: Agri-Food Bio-Residues

The global agri-food sector faces significant challenges in the 21st century, with food security and proper waste management at the forefront. According to the Food and Agriculture Organization of the United Nations, more than one-third of food meant for human consumption is wasted within the supply chain, resulting in financial losses, the depletion of natural resources, and environmental damage. For instance, 25–30% of the 1.4 million metric tons of processed fruits annually turn into waste or by-products, making horticultural residues responsible for 45% of worldwide food waste [22].

Depending on its source, food waste can be divided into two primary groups: vegetable and animal. The dairy, meat, fishery, and seafood processing industries are the main producers of animal-based waste. Depending on the source, vegetable-based bio-residues can include grains, roots and tubers, oil crops and pulses, fruits, and vegetables. The perishable nature of these wastes and logistical difficulties complicates their management. Nonetheless, a significant amount of organic matter that is abundant in lipids, proteins, carbohydrates, and bioactive substances is present in all of these bio-residues [43,44]. Reducing food waste and utilizing it to create value-added products can enhance the efficiency of the food supply chain, lower associated costs, and improve food accessibility and security. Additionally, there is a growing demand for functional and clean-label products driven by modern lifestyle needs. In this context, fruit and vegetable waste holds the greatest potential due to its nature and high production volumes [22].

Among the many factors contributing to the global environmental burden, vegetable food waste has emerged as a significant concern. In Europe, horticultural (fruit and vegetable) processing waste is the fifth largest contributor to overall food waste, accounting for 8% of the total [45]. The discarded fraction in fruit processing industries is typically high, depending on location and harvest methods, with citrus production and processing reaching up to 60%, grapes 20–40%, mangoes 30–50%, bananas 20%, and pomegranates 40–50%, just to name but a few. These fruit wastes, composed of peels, seeds, roots, and pomace, are high in moisture and microbial loads, leading directly to environmental pollution. Processing industries, particularly in developing countries, face financial, spatial, and regulatory constraints regarding waste disposal [22,45].

Among the various strategies proposed for the sustainable management of horticultural waste, valorization stands out prominently. In particular, this approach is driven by the interest in utilizing fruit and vegetable by-products generated during food production, processing, or consumption as an economical and abundant source of high-value bioactive compounds, such as polyphenols, tocopherols, carotenoids, and fatty acids, among others. These compounds can be harnessed as functional food ingredients or nutraceuticals, or employed to produce other valuable bioproducts such as antibiotics, enzymes, biopolymers, and biofuels [3,44,46]. By adopting this biorefinery approach, sustainability is promoted through the reduction of pressure on natural resources and the decreased necessity for landfill waste disposal. Furthermore, the valorization of agri-food residues seamlessly integrates environmental considerations into product development and food production, consistently striving for the highest standards of quality, safety, and efficiency. This approach aligns with the principles of the circular economy and facilitates the achievement of the SDGs, particularly Goal 12: Responsible Consumption and Production [1,2,44].

Regarding the recovery of bioactive compounds from vegetable agri-food bio-residues, polyphenols have already been reported in coffee wastes [47], apple peels [48], pomegranate peels [11], industrial tomato processing waste [14,40], fruit seeds [49,50,51,52], winery wastes [12,15,28,53,54], chestnut waste [55,56], residual brewing yeast [57], and soybean residue (okara) [19], just to name a few. Similarly, plum [3], guava, mango [52], and cherry seeds [35], as well as pear [2] and apple pomace [58] and citrus biomass [59], have been reported to be abundant sources of tocopherols. Regarding fatty acids, once again, the seeds of stone fruits such as apricot and cherry [60], as well as cocoa waste flour [61], chamomile waste [62], and okara [19], have demonstrated high concentrations of oleic, linoleic, and palmitic acids. Further to this, carotenoids have been identified in various agri-food residues, showcasing their potential as valuable bioactive compounds. Notably, tomato residues have been extensively studied [14,63], followed by carrot [64] and pumpkin by-products [46,65] and avocado seeds [66]. Table 1 provides a comprehensive overview of the principal categories of the bioactive compounds aforementioned along with their representative agri-food waste sources.

## 4. Sustainable Extraction Methods of Bioactive Compounds from Bio-Residues

The extraction process is critical for recovering, isolating, and identifying bioactive compounds. However, the diverse physicochemical characteristics of these substances and their matrices make a universal extraction method impractical. Instead, customized approaches must be designed for each specific bioactive compound or compound family and its respective matrix. In addition, multiple factors influence the choice of an extraction methodology. Beyond extraction yield, considerations such as overall cost, product safety, environmental impact, operating time, and scalability for industrial applications are equally important [28].

**Figure 4:**
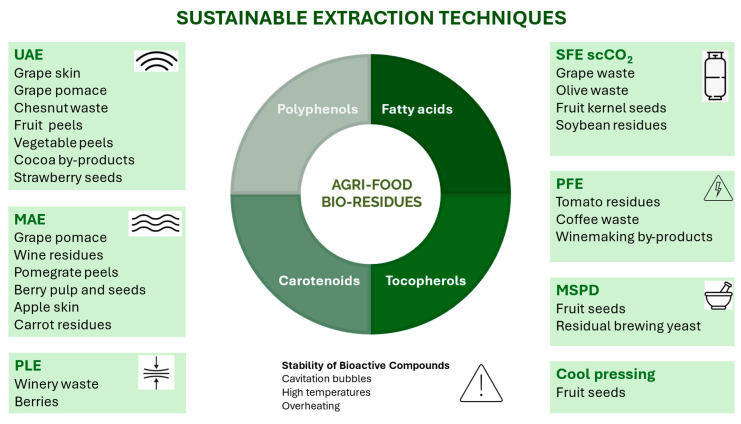
Main extraction techniques for the sustainable recovery of bioactive compounds from agri-food bio-residues.

Extraction technologies, including UAE, MAE, SFE, PLE, PEF, and MSPD, have mainly emerged as sustainable alternatives to conventional solid–liquid extraction (CSLE) traditional methods, like maceration and Soxhlet extraction, for the recovery of bioactive compounds from agri-food bio-residues at both the analytical and industrial levels (Figure 4). These innovative techniques aim to promote faster and more environmentally friendly processes by reducing extraction time, simplifying processing steps, and minimizing energy consumption, solvent usage, and waste generation. However, issues such as overheating of the matrix, leading to loss of activity due to the instability of polyphenols, along with high energy consumption and reliance on organic solvents, may restrict their use and future application [12].

UAE key advantages include reduced extraction time, lower energy and solvent consumption, and improved extract quality and yields compared to conventional maceration. It has been applied for extracting phenolic compounds from a wide variety of waste such as grape skin [73] and grape pomace [12]; Mediterranean fruits and vegetables (apple, peach, cucumber, and red pepper) [74]; chestnut burs, shells, and leaves [10]; tomato, watermelon, and apple peels [75]; cocoa by-products (husk and bean) [27]; mango (peel, endocarp, and kernel) [76]; and date palm residues [77]. Additionally, UAE with ethanol–water mixtures have been employed for the improvement of polyphenol recovery from tomato peel waste. The residues obtained after the application of this UAE protocol were also rich in bioactive fatty acids [40]. Other authors have used deionized water for the sustainable UAE of polyphenols from cherry by-products [50], onion peels [78], saffron tepals [79], and defatted strawberry seeds, obtaining antioxidant and cytoprotective extracts abundant in tiliroside, kaempferol glucoside, and ellagic acid, which could be potentially used as additives for skin care products [49]. Additionally, extracts rich in both polyphenols and carotenoids have been obtained from bael fruit pulp waste using UAE with ethanol [37].

However, when UAE is used, caution is needed, as the generation of cavitation bubbles can produce hydroxyl radicals, potentially leading to the degradation of polyphenols [73].

MAE represents an unconventional technique that can reduce the extraction time and solvent volume compared to CSLE, also resulting in high-quality extracts in terms of composition and biological activity [28]. It has been efficiently used for the extraction of polyphenols from grape pomace [12], bunch stem and cane wine residue samples [28], and pomegranate peels [11]. Buratto et al. have employed MAE and pressurized MAE to recover polyphenols from açaí black-purple berry pulp and seed residues [80]. Apple skins have also been used as a polyphenol source by applying MAE with green solvents such as ethanol aqueous solution [48], and MAE in combination with green or food-compatible solvents was employed for the extraction of carotenoids and polyphenols from carrot-processing rejects [64]. Other authors have used MAE to obtain phenolic-rich extracts from date palm sap and date seeds. Nevertheless, excessive microwave exposure can result in overheating and the decomposition of phenolic compounds, potentially reducing both their bioactivity and extraction yields [77].

Alternatively, SFE is a novel technique to extract bioactive compounds from agri-food bio-residues based on the use of a supercritical fluid that can be easily separated from the extracted product by depressurization [73]. This fluid possesses strong solvating power (in its liquid form), high diffusivity, low viscosity, and minimal surface tension. These properties enable rapid mass transfer and improved penetration into the matrix’s pores, resulting in a more efficient extraction process without leaving chemical residues and offering the potential for automation. Since this technique utilizes compressed fluids, it is more environmentally friendly, as it minimizes the use of organic solvents and enables extraction with non-polluting, non-toxic solvents like water or supercritical CO_2_. Although SFE has demonstrated yields that are equal to or superior to those of conventional methods, it does come with some drawbacks, including high operational costs and the requirement for significant solvent-to-solid ratios when a cosolvent is not utilized. Additionally, the high pressures required for these techniques necessitate specialized and costly equipment, which can sometimes outweigh the technical advantages.

The effectiveness of the SFE method depends on the choice of supercritical fluid. For extracting polyphenols, supercritical carbon dioxide (scCO_2_) is one of the most used solvents [28]. Thus, it has been employed to isolate and purify phenolic compounds and proanthocyanins from grape seed oil and in the recovery of resveratrol from grape pomace [12], stilbenes from cane wine residues [28], polyphenols from grapevine leaves [53], and date palm waste [77]. SFE has also been employed for enhancing polyphenolic compound production from olive waste extracts (pruning biomass, leaf, and exhaust pomace) by using scCO_2_ and 60% ethanol as a cosolvent. The obtained extracts were rich in hydroxytyrosol and chlorogenic, caffeic, and ferulic acids [81]. On the other hand, Casquete et al. have employed scCO_2_ for the extraction of lipophilic substances such as terpenoids, fatty acids, and oxylipins associated with antihypertensive and antimicrobial activities from solid by-products of the wine industry [33]. Both polyphenols and tocopherols have also been obtained from alperujo, a semi-solid residue from the olive oil industry, by means of SFE assisted with ethanol [36]. Furthermore, the effect of extraction conditions on oil yield, total tocopherols, and total carotenes was evaluated by Putra et al. using scCO_2_ assisted by ethanol from palm fiber [82]. In fact, scCO_2_ extraction has been proposed as an alternative to conventional oil sources for obtaining high-quality oil from plum kernel seeds rich in oleic and linoleic acids and tocopherols [83]. Cerón-Martínez et al. have also employed scCO_2_ to obtain bioactive fractions from guava and mango seeds rich in squalene, γ-tocopherol, α-tocopherol, campesterol, β-sitosterol, and stigmasterol [52], while fatty acids and phenolic compounds have been obtained from soybean residue (okara) through scCO_2_ extraction with and without ethanol as a cosolvent [19]. In addition, Aimone et al. have employed subcritical water to develop a protocol tested at pre-industrial levels to isolate the enriched fractions of monomeric polyphenols and tannins from chestnut wood waste [55].

On the other hand, accelerated solvent extraction, also referred to as pressurized fluid extraction (PFE), or PLE, leverages the unique characteristics of conventional solvents under elevated temperatures and high pressures. However, high temperatures may impact the stability of bioactive compounds, potentially leading to reduced extraction yields due to their degradation [73]. Tapia-Quirós et al. evaluated the performance of UAE, MAE, and PLE using ethanol–water mixtures for the recovery of polyphenols from winery waste. The best extraction efficiency was provided by PLE; however, considering not only extraction performance but also investment and operational costs, UAE was proposed for a future scaling-up evaluation [54]. However, compared to SFE using 15% ethanol as a co-solvent, PLE at 100 °C with 50 and 75% ethanol showed higher extract yield and efficiency to recover phenolic compounds, flavonoids, and carotenoids when the residue from pineapple juice processing was employed as a source of bioactive compounds [13]. Furthermore, Huamán-Castilla et al. have demonstrated the potential of hot PLE combined with isopropanol as an environmentally friendly method to selectively recover polyphenols from discarded blueberries [84].

Other recently introduced extraction techniques are electrical treatments such as PFE, which relies on the phenomenon of electroporation. It is regarded as an emerging, non-thermal, ecological, and cost-effective technology that operates over short time frames (microseconds) and with low energy consumption. In PEF, cells are electropermeabilized through the application of an electric field between two electrodes submerged in a solution. This results in the fragmentation of particles and cells, thereby facilitating the extraction of intracellular compounds. PEF treatment can reduce the degradation of heat-sensitive compounds when a moderate electric field is applied. Additionally, because this method is non-destructive, does not produce waste, and allows for rapid processing with low energy consumption, it demonstrates significant viability and potential for industrial implementation. However, PEF widespread adoption is hindered by the high costs associated with implementation [12]. This technology has been applied to recover polyphenols from winemaking by-products, including grapes, grape pomace, and vine sprouts [12]. In addition, it has been used for the waste valorization of industrial tomato processing. Thus, PEF was applied to three different steps, allowing for the extraction of high-added-value compounds, such as carotenoids and phenolic compounds, from juicing residues [14].

Additionally, other extraction methods have been proposed to obtain bioactive compounds from agri-food bio-residues. Thus, MSPD has been efficiently applied to the extraction of polyphenols from pressed grape seeds [15] and grape residues [85], residual brewing yeast [57], defatted cherry seeds obtained after cherry liquor manufacturing [51], defatted plum seed by-products [3], and sloe residues [86]. This extraction method involves mechanically mixing the sample with an abrasive, inert material, followed by eluting the analytes with a suitable solvent [15]. To increase selectivity and cleaning efficiency, MSPD is assisted by TiO_2_ nanoparticles, which interact with phenolic compounds through their diol groups, in a similar way to chelation [85]. MSPD offers several advantages over traditional extraction methods, such as operation at room temperature and atmospheric pressure, reduced solvent consumption, low energy and equipment demands, and straightforward, rapid, and simple procedures. Furthermore, it allows for simultaneous extraction and purification, ensuring high recovery rates of polyphenols while maintaining the functional properties of the extract. As a result, MSPD is regarded as an efficient and sustainable approach for producing phenolic-rich extracts [15,85]. However, this method is challenging to apply at an industrial scale due to the complexity of scaling up the process and the need for precise control over extraction conditions, which make it difficult to implement MSPD efficiently in large-scale operations.

In recent years, hybrid extraction technologies have emerged, as combining two techniques can improve the overall process by offsetting each method’s limitations. While synergistic effects have been observed, leading to higher extraction yields and improved selectivity, challenges related to cost-effectiveness, energy efficiency, and process scalability may restrict the widespread application of these hybrid systems. As such, PEF has been integrated with MAE to establish a green protocol for recovering polyphenols from coffee waste, offering short processing times and minimal reagent usage. This approach presents a compelling enhancement to coffee biorefinery strategies [47]. Furthermore, the combined use of PEF and US in pretreatment processes has also been applied to the hydroalcoholic extraction of polyphenols, ascorbic acid, hesperidin, and carotenoids from orange peels [87]. Additionally, the hybrid combination of US and MAE (USMAE) has been investigated for the extraction of polyphenols from mango peels [88] and as a pretreatment approach to improve anthocyanin extraction from grape pomace using natural deep eutectic solvents (NADESs) [89].

On the other hand, bioactive fatty acids, tocopherols, and carotenoids have been successfully recovered from various agri-food residues from the previous extraction of oils. Traditionally, the conventional Soxhlet method has been revered as the gold standard, owing to its straightforwardness and superior efficiency in extracting fat-soluble fractions. Among the solvents, *n*-hexane is predominantly favored because of its excellent oil solubility, affordability, high volatility, low boiling point, and ease of removal from solids. Thus, Rodríguez-Blázquez et al. have valorized peach, apricot, plum, and cherry seeds by obtaining oils rich in oleic and linoleic acids with heart-healthy lipid indexes and high oxidative stability [60]. Similarly, oils from plum seeds rich in unsaturated fatty acids, mainly oleic acid and α-, γ-, and δ-tocopherols, have been obtained [3], as well as oils rich in SFA and unsaturated or omega fatty acids from *citrus* seeds [67]. The total lipid content and total carotenoids (sum of β-carotene and lycopene) from tomato residues have also been determined in hexanoic extracts obtained with Soxhlet [63].

Nevertheless, the challenges associated with the use of toxic hexane have led to the emergence of cold pressing in recent years as a green and sustainable alternative to traditional Soxhlet extraction, free from any chemical contaminants. This method has been particularly effective in obtaining oils rich in α-tocopherol, linoleic acid, and palmitic acid from lemon seeds [90]. Vladić et al. also proposed cold pressing as an alternative to conventional oil sources to obtain high-quality oil from plum kernel seeds rich in oleic and linoleic acids and tocopherols [83]. In addition, oil from fresh sour cherry kernels, characterized by linoleic, oleic, *α*-eleostearic, and palmitic acids, was obtained using the cool press method [91].

Alternatively, a hydro-distillation process has also been employed for the extraction of essential oil rich in fatty acids, especially linoleic acid, from German Chamomile agri-waste [62]. Other authors have explored the use of vegetable oils, such as canola, corn, and soybean oils, as eco-friendly alternatives to traditional organic solvents for extracting carotenoids from dried pumpkin pulp waste. This approach yields an oil naturally enriched with carotenoids, which holds potential as a functional ingredient for applications in food, cosmetics, and medicinal product development [46].

Moreover, anaerobic fermentation has been performed as an alternative to conventional and unconventional extraction methods for the recovery of volatile fatty acids from food waste saccharified residues, demonstrating its potential for n-butyrate production at a low cost [92].

In the realm of solvent selection for the extraction of bioactive compounds from agri-food waste, organic solvents like methanol, ethanol, or hexane have historically been favored for their high efficiency and cost-effectiveness [48,60,80,84,86]. Nevertheless, the utilization of organic solvents, even in minimal quantities, is increasingly frowned upon in sectors such as food, cosmetics, and pharmaceuticals. Consequently, there is a burgeoning trend towards adopting more sustainable solvents that aim to supplant volatile solvents with biocompatible and eco-friendly alternatives. Noteworthy examples of these environmentally friendly solvents include bio-based options such as ethyl alcohol, ethyl acetate, ethyl lactate, supercritical carbon dioxide, diethyl carbonate, and deep eutectic solvents [93]. In this context, the use of NADESs has also been proposed as a greener extraction alternative. They represent a new generation of liquid salts, typically composed of inexpensive and readily available components such as non-toxic quaternary ammonium salts (e.g., cholinium chloride) and naturally derived, uncharged hydrogen-bond donors (e.g., amines, sugars, alcohols, and carboxylic acids). These solvents, when combined with the aforementioned non-conventional techniques (e.g., UAE, MAE) or even with CSLE, offer a more sustainable approach to the extraction of bioactives from residues. Some suggested applications of NADEs involve their use as extractants with UAE to obtain polyphenols from grape pomace, skins, and seeds, especially those formulated with choline chloride [94,95]. Other authors have used eutectic solvents composed of choline chloride and polyols, combined with MAE under relatively mild conditions, to sustainably extract polyphenols from vine pruning residues [96]. Deep eutectic solvents (DESs) synthesized using choline chloride with various hydrogen bond donors such as malic acid, glycerol, ethylene glycol, and urea, as well as a benchmark solvent ethanol-water 50:50 (*v*/*v*), have been effectively employed to obtain bioactive extracts rich in catechin, epicatechin, rutin, syringic acid, and *p*-coumaric, chlorogenic, and ferulic acids from apple peels under a conventional reflux method of condensation [97]. Combinations of choline chloride-based DESs with an acid, a polyol, or a sugar as hydrogen bond donors have also been used for the extraction of polyphenols from chestnut shell waste. The recyclability of the DES has also been proven, which could make the extraction process viable and amenable for large-scale applications [56]. Additionally, Gaharwar et al. recovered polyphenols from pomegranate peels using choline chloride as a hydrogen bond acceptor and ethylene glycol, glycerol, malic acid, and urea as hydrogen bond donors [98].

Conversely, supramolecular biosolvents (bioSUPRAS), produced through energy-efficient and eco-friendly processes using low-toxicity ingredients, have recently been applied to recover phenolic compounds from orange peel waste generated by the tea industry. These bioSUPRAS present a promising alternative for obtaining extracts suitable for formulations in the cosmetic, nutraceutical, and food industries [99].

## 5. Characterization of Bioactive Compounds in Bio-Residue Extracts

### 5.1. Determination of Polyphenols

The determination of polyphenol content in bio-residues is crucial for evaluating their potential health benefits and for the development of potential applications [100]. There are two primary approaches to polyphenol determination: total content and phenolic profile analysis. The determination of total content is generally less specific and is commonly performed using spectrophotometric methods. These methods, such as the Folin–Ciocalteu assay, are relatively simple and quick but do not differentiate between individual polyphenolic compounds and can be influenced by other reducing substances present in the sample.

In contrast, the determination of the phenolic profile involves the identification and quantification of individual polyphenolic compounds. This is typically achieved using high-performance liquid chromatography (HPLC) coupled with various detectors, such as DADs or MS. These methods allow for the separation of complex mixtures of polyphenols, providing detailed information on the specific types and quantities of polyphenols present in the sample. This is more specific and sensitive compared to spectrophotometric assays but it requires more sophisticated techniques and expertise.

Only 58% of research papers that determine the TPC simultaneously determine the phenolic profile [17]. Moreover, there are another 13% of research papers that study the phenolic profile without reporting the TPC data [2]. This indicates that there is still much to be done to deepen the understanding of the phenolic composition of agri-food bio-residue extracts since without a detailed phenolic profile, it is challenging to identify the specific phenolic compounds responsible for the observed bioactive properties. This is in addition to the fact that TPC overestimates the real phenolic content since other reductant substances can interfere. Therefore, a comprehensive analysis that includes both TPC and the phenolic profile is crucial for a thorough understanding of the bioactive potential of agri-food bio-residues. This approach can lead to better utilization of these residues in developing functional foods, nutraceuticals, and other health-related applications.

#### 5.1.1. Total Phenolic Content Determination

Spectrophotometric methods for determining the TPC, or specific subclasses thereof, do not provide as much detailed information as chromatographic methods for determining the phenolic profile. However, they remain widely used due to their simplicity and low cost, offering complementary results that aid in interpreting the origin of the bioactive properties exhibited by agri-food bio-residues.

The Folin–Ciocalteu assay is a widely employed method for quantifying the TPC in various samples, including plant extracts and food residues [48]. This method is based on the reduction of the Folin–Ciocalteu reagent (a mixture of sodium tungstate and sodium molybdate in phosphoric acid) by phenolic compounds, resulting in a color change that can be measured spectrophotometrically. The procedure involves adding phosphomolybdotungstic acid (yellow color), which is reduced by the phenolic groups, generating a blue complex [80]. After an incubation period, the absorbance is measured at 765 nm. TPC is quantified using a calibration curve with a known standard, such as gallic acid, and is expressed in gallic acid equivalents (GAE) [101]. However, on some rare occasions, it can be expressed as catechin equivalents (CE) [17]. This method is valued for its simplicity and accuracy, although it may be influenced by the presence of other reducing substances.

For the total flavonoid content (TFC) determination, the extract is usually dissolved in MeOH or EtOH, followed by the reaction with AlCl_3_, which favors the formation of a complex between aluminum and the flavonoids present in the extract. After 30 min incubation, absorbance is measured at 415 nm using UV–Vis spectroscopy, employing a calibration curve of a reference flavonoid compound. This method is employed in 48% of the research papers that study the TPC, among those included in this section. Nevertheless, there is no consensus on the reference flavonoid with which to report this value. The results are frequently expressed as quercetin (QE) (31%) [62], rutin (RE) (25%) [29], or catechin (19%) [102] equivalents. However, some authors reported TFC data as gallic acid (6%) [80], epicatechin (6%) [11], or even trolox (TE) (6%) [103] equivalents, or did not report the equivalence compound used (6%) [104].

Total flavone content, also known as yellow flavonoids, can be determined by dissolving a small amount of the solid extract in acidified ethanol (15% HCl 1.5 M), measuring absorbance at 374 nm employing UV–Vis spectroscopy [105]. This method is only applied in 3% of the research papers that studied TPC.

Total flavonol (TF) content is usually determined by diluting the extract solution in an ethanolic solution previously acidified with 2% HCl. Absorbances of samples are measured at 360 nm and results are usually expressed as QE [106]. This method is only applied in 3% of the research papers that studied TPC.

The vanillin assay is a colorimetric method used to quantify flavan-3-ol content in various samples. This assay involves the reaction of vanillin with flavan-3-ols in an acidic medium, forming a colored complex. The absorbance of this complex is measured at approximately 500 nm using UV–Vis spectroscopy. The flavan-3-ol content is then calculated using a calibration curve with a known standard, such as catechin, expressing the final result as CE [27]. This method is only applied in 3% of the research papers that studied TPC.

The pH differential method is a widely used technique for quantifying total anthocyanin content (TAC) in various samples. This method leverages the color change of anthocyanins at different pH levels. The samples are diluted in buffers at pH 1.0 and pH 4.5, and their absorbance is measured at 520–535 nm and 700 nm using a spectrophotometer [105]. The difference in absorbance at these wavelengths is used to calculate the TAC, considering factors such as molecular weight and molar extinction coefficient. This method is both rapid and accurate, allowing for the effective quantification of anthocyanins even in the presence of interfering compounds. The results are usually expressed as cyanidin [80] or cyanidin-3-O-glucoside [17] equivalents. This method is applied in 9% of the research papers that studied TPC.

The total tannin content can be measured by the difference between the TPC and the content of simple phenolics [105]. To achieve this, bovine serum albumin (BSA) is commonly used to facilitate the binding of tannins, which helps in the separation of tannins from other phenolic compounds. The mixture is then centrifuged, which allows for the tannin–BSA complexes to precipitate. The supernatant, which contains the simple phenolics, is carefully collected and analyzed to determine the content of simple phenolics using the Folin–Ciocalteu reagent in an identical way to the TPC method. This difference gives the total tannin content, expressed in tannic acid equivalents (TAE). This method is only applied in 3% of the research papers that study TPC. However, instead of a BSA reagent, cinchonine hemisulfate can also be used to determine total tannin content. After the addition of this reagent to the extract solution, the mixture is vortexed and allowed to react overnight. Following the reaction, the mixture is centrifuged to precipitate the tannin–cinchonine hemisulfate complexes. In a similar way to the BSA assay, the difference between the Folin–Ciocalteu value of the supernatant, which contains the simple phenolics, and the TPC, allows for the determination of the total tannin content [55].

The condensed tannin content can be quantified using the butanol–HCl assay since the intermolecular bonds of proanthocyanidins are cleaved under acidic conditions, releasing anthocyanidins that absorb at 550 nm. Briefly, the extract is mixed with butanol-HCl and ferric ammonium sulfate, heated in a water bath, and allowed to cool at room temperature. The absorbance is measured at 550 nm by means of UV–Vis spectroscopy and the condensed tannin content is expressed as CE [107].

The hydrolyzable tannin content can be determined with a nitrous acid test. Following the hydrolysis of the extract with hot dilute sulfuric acid, the mixture is allowed to react with sodium nitrite, and the absorbance is measured at 538 nm immediately and 30 min later. The content of ellagic acid and ellagitannins is directly proportional to the increase in absorbance at 30 min. A basic rhodanine test under the presence of KOH and the following measure of absorbance at 520 nm can also be applied to hydrolyze the extracts to determine the presence of gallotannins [108].

Total hydroxycinnamic acid (THA) content is frequently determined spectrophotometrically by dilution with 2% HCl ethanolic solution, measuring the absorbance of extracts at 320 nm, and the THA content is expressed generally as caffeic acid equivalents (CAE) [106]. This method is only applied in 3% of the research papers that studied TPC.

In addition to the aforementioned quantitative methods, several qualitative assays can indicate the presence of specific compound families [98]. The acid test involves adding a few drops of diluted sulfuric acid to an aliquot of the extracts, where the presence of flavonoids is indicated by the apparition of an orange color. The ferric chloride test entails adding a few drops of 5% FeCl_3_ solution to the extracts, with the presence of tannins being indicated by the formation of a blue-black color. Lastly, the lead acetate test involves adding some drops of 10% lead acetate solution, where the presence of tannins is shown by the formation of a yellowish precipitate.

#### 5.1.2. Individual Phenolic Content Determination

The identification and quantification of the phenolic profile provide indispensable information for understanding the bioactive potential of agri-food bio-residues, as each phenolic family can exhibit different health effects. Therefore, a thorough study should not be undertaken without offering information on the determination of individual polyphenols. As previously mentioned, only 58% of the studies reporting TPC simultaneously evaluate the phenolic profile.

Among the techniques employed for this evaluation, LC stands out as the predominant separation method used to isolate phenolic compounds for subsequent detection and quantification. Of the research papers utilizing this technique, 74% employed HPLC [17] and 26% used ultra-high-performance liquid chromatography (UHPLC) [29]. These methods are primarily conducted in reverse phase (RP) utilizing a non-polar stationary phase, mainly C18 [3]. Common aqueous mobile phases include the presence of additives such as 0.1% [109] or 5% [16] formic acid (HF), 5 mM ammonium formate [98], or 2% acetic acid [105], for example. Commonly employed organic mobile phases are based on acetonitrile (ACN) [3] or MeOH [105] without additives, as well as ACN or MeOH with additives such as 0.1% HF [106] or 5 mM ammonium formate [98]. The use of gradient elution is observed in almost all the research papers studied [106]. The general flow ranged from 0.25 mL·min^−1^ in UHPLC columns [29] to 1 mL·min^−1^ in conventional HPLC columns [105]. The analysis time in the obtained chromatograms ranges from 8 min in UHPLC [29] to up to 55 min in HPLC [109] for the separation of phenolic compounds present in agri-food bio-residues, depending on the sample profile.

After chromatographic separation, MS is used in 35% of the analyzers to evaluate the phenolic profile of extracts with bioactive properties [3]. Within this category, 63% are single MS analyzers [27] and 37% are tandem MS (MS/MS) analyzers [106]. Among the single MS analyzers, time-of-flight (TOF) analyzers are predominant (80%) [109] compared to quadrupole (Q) analyzers (20%) [17]. For MS/MS analyzers, triple quadrupole (QqQ) analyzers are the most common (67%) [98], followed by quadrupole time-of-flight (QTOF) analyzers (33%) [106]. ESI stands out as the main source of ionization across all of these studies. A negative ionization mode is generally employed for the determination of phenolic compounds [27], although a positive ionization mode is sometimes used [109]. The mass-to-charge ratio (*m*/*z*) range studied extends from 20 [109] or 50 [106] *m*/*z* to 1200 [106] or even 1500 [5] in some cases, depending on the phenolic compounds expected to be found in a given agri-food bio-residue. The fragmentation energy used to obtain fragments in MS/MS depends on the structure of the precursor ion and the specific fragment, ranging from 4 eV [106] to 100 eV [5] in some cases.

Another 35% of the analyzers are DADs [110], selecting a wavelength between 200 and 400 nm, which may depend on the subclass of polyphenols being studied [109]. Common wavelengths used are 240–290 nm for flavonoids [105] and 300–330 for phenolic acids [16], for example. DADs are also used in conjunction with MS (35%) [16]. Among the MS analyzers coupled with DADs, linear ion trap (50%) [58], orbitrap (37%) [5], and quadrupole (13%) [102] analyzers are the main ones.

#### 5.1.3. Polyphenol Content in Agri-Food Bio-Residue Extracts

This section summarizes the most relevant results concerning the phenolic content of extracts from agri-food bio-residues, as observed in the most illustrative research papers found in the literature. These studies report both TPC and their subclasses using spectrophotometric methods, as well as the phenolic profile determined through chromatographic methods (Table 2).

Rodríguez-Blázquez et al. reported the TPC, TFC, and phenolic profile of plum (*Prunus domestica* L.) seed residue extracts [3]. The TPC values ranged from 0.57 to 2.40 mg GAE per gram, being higher when the residue was subjected to higher temperatures. TFC values were 0.9–1.3 mg QE per gram, while the authors reported that this result was higher than other studies of plum seeds within this variety. The phenolic profile was acquired using HPLC–QTOF–MS/MS, identifying seventeen different polyphenols, belonging to hydroxybenzoic acids (5), hydroxycinnamic acids (5), and flavanols (7).

Santos et al. evaluated the TPC, total flavones, TAC, total tannin content, and phenolic profile of acerola (*Malpighia emarginata*) residue extracts [105]. The TPC value was 9.0 mg GAE per gram of dry matter, total flavones were 3.3 g·g^−1^ of dry matter, TAC was 0.57 g·g^−1^ of dry matter, and the total tannin content was 0.35 mg TAE per gram of dry matter. The phenolic profile was evaluated using HPLC–DAD, obtaining 2.05 mg of phenolic compounds per g of dry matter. This result evidenced the overestimation of the TPC method, being four times lower than the latter. Sixteen different phenolic compounds were identified, mainly phenolic acids (10) and flavonoids (6).

Buratto et al. reported the TPC, TFC, and TAC of açaí (*Euterpe oleracea Mart.*) residues (seeds, slurry, and pulp) but without reporting the phenolic profile [80]. The TPC value was higher for pulp (28.6 mg GAE·g^−1^ of dry matter) than for seeds (17.7 mg GAE·g^−1^ of dry matter) and slurry (1.6 mg GAE·g^−1^ of dry matter). Regarding TFC values, the highest content was found for pulp (2.53 mg GAE·g^−1^ of dry matter) and seeds (2.42 mg GAE·g^−1^ of dry matter), with slurry extracts being the ones with the lowest flavonoid content (0.82 mg GAE·g^−1^ of dry matter). TAC could only be detected in pulp residues (2.93 mg·g^−1^ of dry matter). However, additional information on the individual phenolic content is needed to better understand their bioactive properties.

Christou et al. evaluated the TPC, TFC, TF, THA, and phenolic profile of taro (*Colacasia esculenta* L.) leaves [106]. TPC values in the extract ranged from 5.0 to 24.0 mg GAE·g^−1^, TFC from 2.5 to 6.0 mg CE·g^−1^, TF from 4.0 to 8.0 mg QE·g^−1^, and THA from 2.0 to 4.5 mg CAE·g^−1^. Individual phenolic content, evaluated with UHPLC–QTOF–MS/MS, allowed for the identification of 12 polyphenols, mainly flavones (eight), flavonols (three), and a caffeic acid derivative (one).

Gaharwar et al. reported the TPC, test for flavonoids, test for tannins, and phenolic profile of pomegranate (*Punica granatum*) peel residues [98]. TPC values ranged from 3.5 to 10 g GAE·L^−1^ of extract, and several qualitative tests indicated the presence of both flavonoids and tannins. An analysis using HPLC–QqQ–MS/MS demonstrated the presence of six major polyphenols (ellagic acid, gallic acid, ferulic acid, p-coumaric acid, protocatechuic acid, and caffeic acid), with ellagic acid being the most abundant.

Tozzi et al. also aimed to investigate the TPC and the phenolic profile of pomegranate (*Punica granatum*) peel by-product [16]. The TPC varied across the different varieties of pomegranates, with values from 115 to 249 mg GAE·g^−1^ of dry weight. Individual polyphenol content, evaluated with HPLC–DAD–MS^n^ employing a linear ion trap, showed the presence of two phenolic acids (coumaric acid derivative and ellagic acid) and eight ellagitannins (α and β-punicalagins accounted for more than 65% of total ellagitannins, which provides this residue with high antioxidant activity).

Cádiz-Gurrea et al. studied the TPC, total flavan-3-ol, and phenolic profile of Peruvian cocoa (*Theobroma cacao*) by-products [27]. The TPC value ranged between 4.9 and 31.3 mg GAE·g^−1^ of dry extract, while the total flavan-3-ol content was between 19 and 130 mg CE·g^−1^ of dry extract. The individual polyphenol content was determined with HPLC–TOF–MS, identifying 49 total compounds. These polyphenols belonged mostly to flavonoids family (29), while some of them were phenolic acid derivatives (five), amino acid derivatives (seven), and other polar compounds (eight).

Albuquerque et al. reported the phenolic profile of Brazilian berry (*Eugenia brasiliensis* Lam.) waste (seeds, peels, and residue), without reporting the TPC value employing Folin–Ciocalteu’s assay [5]. Using HPLC–DAD–Orbitrap–MS, the authors stated that hydrolyzable tannin derivatives were the predominant phenolic compounds. Seventeen hydrolyzable tannin derivatives were identified in berry seeds (104 mg·g^−1^ of extract) and berry residue (85 mg·g^−1^ of extract), while only 13 compounds were present in berry peels (13 mg·g^−1^ of extract). On the other hand, seven condensed tannins were also detected, with the highest concentration found in berry peel (27 mg·g^−1^ of extract). From the flavonoid family, three quercetin derivatives were detected (C_peels_ = 17 mg·g^−1^ of extract; C_residue_ = 7.2 mg·g^−1^ of extract; C_seeds_ = 5.8 mg·g^−1^ of extract). From the anthocyanin family, four compounds were identified, with the peels showing the highest concentration (77 mg·g^−1^ of extract), followed by the residue (17.5 mg·g^−1^ of extract), and the seeds (12.6 mg·g^−1^ of extract). The authors stated that due to the high amount of anthocyanins present in the peel extract, it could provide multiple benefits regarding cognitive function and memory while preventing cardiovascular diseases and some types of cancer. Total phenolic compounds, excluding anthocyanins, were 115 mg·g^−1^ of extract for the seeds, 101 mg·g^−1^ of extract for the residue, and 57 mg·g^−1^ of extract for the peels.

Del Castillo-Llamosas et al. studied the TPC, TFC, and phenolic profile of avocado (*Persea americana*) peel waste extracts, obtained at six different temperatures (140–180 °C) [29]. The TPC values were between 31.3 and 40.6 mg GAE·g^−1^ of dry extract, being at a maximum for the extract obtained at 170 °C. Moreover, TFC values ranged from 61.3 to 70.5 mg RE·g^−1^ of dry extract, which was also the highest for the extract obtained at 170 °C. Using UHPLC–TOF–MS, the authors tentatively identified more than 35 different compounds (mainly flavonoids, phenolic acids, and lignans) in the ethyl acetate extract of avocado peel waste. Among phenolic acids, the hydroxybenzoic acid subclass was the most representative, although hydroxycinnamic acids were also detected. Several subclasses of flavonoids were identified, such as flavonols, flavones, and flavanones, which undoubtedly provided this extract with important health benefits.

Polaki et al. analyzed pineapple (*Ananas comosus* L.) fruit and peel wastes in order to evaluate the TPC, TFC, and phenolic profile in these waste products [109]. The methanolic extract from peel and fruit waste presented higher TPC values (54.8 and 42.7 mg·g^−1^, respectively) than hexane (22.7 and 16.6 mg·g^−1^, respectively) and aqueous extracts (15.0 and 14.4 mg·g^−1^, respectively). Likewise, the highest TFC values were obtained for peel and fruit waste methanolic extracts (49.7 and 41.6 mg·g^−1^, respectively), followed by hexane (41.1 and 32.8 mg·g^−1^, respectively) and aqueous extracts (35.3 and 26.9 mg·g^−1^, respectively). An analysis using HPLC–DAD–TOF–MS, showed the presence of 17 phenolic compounds for pineapple peel waste extracts (cinnamic, 18.26 mg·L^−1^; caffeic, 13.08 mg·L^−1^; and p-hydroxybenzoic acid, 10.82 mg·L^−1^ being the main ones) and only 12 polyphenols for fruit waste extracts (where cinnamic acid, 14.25 mg·L^−1^, presented the highest concentration). These polyphenols belonged to the hydroxybenzoic and hydroxycinnamic acid subclasses.

These results highlight the variability in the total phenolic content and phenolic profiles of agri-food bio-residues, determined with spectrophotometric and chromatographic methods. Studies reveal that phenolic content varies depending on the type of residue and extraction conditions, identifying numerous specific phenolic compounds, such as phenolic acids, flavonoids, lignans, and tannins. These findings underscore the potential of agri-food residues as valuable sources of bioactive compounds.

### 5.2. Determination of Tocopherols

The determination of tocopherols in agri-food bio-residues is of paramount importance due to their potent antioxidant properties and potential health benefits. Tocopherols are commonly found in various agri-food residues such as seeds, peels, and pulp, being practically determined using chromatographic methods (Table 3).

Advanced analytical techniques, including HPLC and GC, are employed to separate these compounds. GC is normally employed in a split (1:10) mode [52], with helium at 1 mL·min^−1^ as a carrier gas [59], and sometimes a derivatization is needed [59]. While GC is frequently coupled with MS analyzers [52], HPLC is often coupled with DADs [3], fluorescence [2], or even MS [35] to accurately quantify and profile tocopherols in these residues. However, RP–HPLC has been reported to be unable to differentiate between β- and γ-tocopherols [3], with only a few exceptions [2]. Consequently, normal-phase HPLC columns, such as polyamide ones, are required to achieve the accurate separation and quantification of these tocopherol homologs [112]. Common aqueous phases are constituted with 0.1% HF [35] or phosphoric acid at pH 3 [36]. Organic phases that are frequently used are MeOH [2], ACN [36], or ACN with 0.1% HF [35]. Flow rates could be up to 1 mL·min^−1^ for HPLC [3] and 0.4–0.45 mL·min^−1^ for UHPLC columns [3], with chromatogram times of less than 9 min for the latter [2]. DAD wavelengths for quantitation are around 290 nm, although others such as 305 nm could also be used [3]. Fluorescence detection requires excitation at 290 nm [58] while measuring the emission at 330 nm [36]. In MS detection, ranges between 45 [59] and 1200 *m*/*z* [59] are frequently screened.

Rodríguez-Blázquez et al. reported the presence of α and δ-tocopherol in plum seed residue using HPLC–DAD [3]. Moreover, the authors stated that it was not possible to differentiate between β- and γ-tocopherol, although a peak at their retention time was detected. The reason was their coelution due to chemical structure similarities under tested conditions. The authors suggested that based on the literature, a more polar stationary phase like pentafluorophenylsilica could be more selective. The combined β- and γ-tocopherol was the predominant homolog (5.7–11.2 mg·kg^−1^), followed by α- (2.0–2.5 mg·kg^−1^) and δ-tocopherol (1.5–2.2 mg·kg^−1^). Fermentation and distillation processes of plum significantly altered the tocopherol content. For instance, α- and δ-tocopherols increased in concentration during fermentation, whereas distillation did not affect their levels. Given that β-tocopherol is minor compared to γ-tocopherol, the γ–α-tocopherol ratio was calculated for all extracts, ranging from 2.27 to 5. This ratio highlighted the relative abundance of γ-tocopherol in the extracts.

Dauber et al. compared two alperujo residues (Coratina and Arbequina) in terms of tocopherol content using HPLC coupled to a fluorescence detector [36]. The Coratina cultivar presented more content of total tocopherols (345–454 mg·kg^−1^) than Arbequina extracts (232–274 mg·kg^−1^). Additionally, α-tocopherol was the most abundant isomer for both Coratina (257–335 mg·kg^−1^) and Arbequina (173–226 mg·kg^−1^) extracts. δ-tocopherol was the minor homolog, with values between 20 and 25 mg·kg^−1^ and 17 and 24 mg·kg^−1^ for Coratina and Arbequina, respectively. However, the authors could not differentiate between β- and γ-tocopherol, whose values were in the range of 63–95 mg·kg^−1^ and 27–43 mg·kg^−1^ for Coratina and Arbequina, respectively.

Juriene et al. evaluated the effect of supercritical CO_2_ extraction of tocopherols in pitted sour cherry pomace using UHPLC–QTOF–MS/MS [35]. Hexane extracts presented lower total tocopherol content (81.8–205.8 µg·g^−1^) than supercritical fluid CO_2_ extracts (141.9–432.0 µg·g^−1^). α-tocopherol presented a higher concentration (81.4–195.9 µg·g^−1^), being more than 50% of total tocopherol content, followed by β + γ-tocopherol (45.6–272.0 µg·g^−1^) and δ-tocopherol (11.4–27.9 µg·g^−1^). However, the authors could not separate β- and γ-tocopherol contributions using this technique. The use of this SFE method improved the content of tocopherols in this type of agri-food bio-product.

Ferreira et al. reported the tocopherol content of pear pomace flours employing UHPLC coupled to a fluorescence detector [2]. The total tocopherol content of this residue was between 468.7 and 913.4 µg·100 g^−1^ of dry weight. Among them, α-tocopherol was the most abundant (341.8–582.5 µg·100 g^−1^ of dry weight), representing almost 80% of the total tocopherol content. Additionally, the authors achieved a complete separation of β- (53.7–83.6 µg·100 g^−1^ dry weight) and γ-tocopherol (37.8–60.6 µg·100 g^−1^ dry weight), δ-tocopherol being the less abundant in this sample (3.0–12.6 µg·100 g^−1^ dry weight). Therefore, pear pomace flours are a great source of tocopherol homologs, especially α-tocopherol.

Brandao et al. analyzed apple pomace from craft cider in terms of tocopherol content using HPLC coupled with fluorescence detection [58]. However, the authors only detected α-tocopherol at 0.4 mg·100 g^−1^ of dry weight, being unable to detect any of the other tocopherol homologs.

Albuquerque et al. evaluated the difference in tocopherol content in Brazilian berry (*Eugenia brasiliensis* Lam) waste belonging to different parts—whole residue, seeds, and peels—using HPLC coupled with a fluorescence detector [5]. Peel extracts (3.56 mg·100 g^−1^ dry weight) presented higher tocopherol content than whole residue (1.62 mg·100 g^−1^ dry weight) and seed (0.97 mg·100 g^−1^ dry weight) extracts. Only α- and γ-tocopherols were identified. While α-tocopherol was the main homolog in peel extract (2.58 mg·100 g^−1^ dry weight), γ-tocopherol predominated in seed (0.74 mg·100 g^−1^ dry weight) and whole residue (1.00 mg·100 g^−1^ dry weight) extracts. These findings suggest that peel extract from Brazilian berry waste is a valuable source of α-tocopherol.

Cerón-Martínez et al. evaluated the tocopherol content in guava (*Psidium guajava*) and mango (*Mangifera indica* L.) seeds using GC–MS without derivatization [52]. While guava seed extracts presented both α- (0.1–0.7 mg·g^−1^) and γ-tocopherols (0.5–2.0 mg·g^−1^), mango seed extracts only showed the presence of α-tocopherol (0.1–0.6 mg·g^−1^). Therefore, both residues had the same amount of α-tocopherol, while guava seeds were also a good source of γ-tocopherol.

Marques et al. studied the tocopherol and tocopheryl ester content in oat (*Avena sativa* L.) straw residue using GC–MS [113]. The total content for tocopherol and tocopheryl esters was between 26 and 71 mg·kg^−1^. γ-tocopherol was the most abundant (4–7 mg·kg^−1^), although δ-tocopherol was also identified (1 mg·kg^−1^). Among tocopheryl esters found, *α*-tocopheryl tetradecanoate was the most abundant (8–24 mg·kg^−1^), followed by *β*-tocopheryl tetradecanoate (5–17 mg·kg^−1^) and *α*-tocopheryl dodecanoate (5–18 mg·kg^−1^). No presence of γ-tocopheryl esters was reported.

Lamine et al. evaluated the tocopherol content in citrus residual biomass using GC–TOF–MS after derivatization with N-methyl-N-trimethylsilyltrifluoroacetamide [59]. Only α- and γ-tocopherol were identified, with α-tocopherol being the most abundant (0.91–2.90 mg·g^−1^) and γ-tocopherol having a minor concentration (0.39–0.60 mg·g^−1^). *C. reticulata* was the citrus species with more tocopherol content. The authors suggest that these extracts could be applied in skin care and cosmetics due to their fragrance and antioxidant benefits.

Tocopherols are ubiquitously present in various agri-food residues, necessitating advanced analytical techniques such as HPLC and GC for their precise separation and their subsequent quantification. The differentiation between β- and γ-tocopherols remains a significant challenge, often requiring more polar stationary phases for effective separation. Fermentation and distillation processes can markedly alter the tocopherol content in these residues, underscoring the importance of processing conditions. Additionally, residues from different crops and fruits exhibit variations in tocopherol content and profiles, highlighting their potential as valuable sources of natural antioxidants for industrial and cosmetic applications.

### 5.3. Determination of Carotenoids

The carotenoids present in the extracts obtained from agri-food waste have been frequently determined as total carotenoids. Spectrophotometric methods based on those proposed by Anton et al. [114] have been employed to determine the total carotenoid content, expressed as the sum of β-carotene and lycopene in tomato residues [63]. The total carotenoids have been also estimated spectrophotometrically according to the method of Lichtenthaler and Wellburn [115], measuring the absorbance of tomato waste extracts at 470 (A_470_), 663 (A_663_), and 647 nm (A_647_). The content of total carotenoids, chlorophyll *a*, and chlorophyll *b* was calculated using the following Lichtenthaler equations [14]:

*Chlorophyll a* = 12.25⋅A_663nm_ − 2.79⋅A_647nm_

*Chlorophyll b* = 21.50⋅A_647nm_ − 5.10⋅A_6663nm_

*Total carotenoids* = (1000⋅A_470nm_ − 1.82*⋅Chlorophyll a* − 85.02*⋅Chlorophyll b*)/228

Furthermore, de Andrade Maia et al. determined the β-carotene content from pineapple waste extracts [13] using the method described by de Rosso and Mercadante [116], with slight modifications, by measuring the absorbance at 450 nm (Table 3). The total carotenoid content was also estimated spectrophotometrically in extracts from carrot industry residues [64], as well as from avocado seeds [66], bael fruit pulp waste [37], and the peel, flesh, and seeds of pumpkin [65], as well as pumpkin byproducts [46], at 450 nm, using a standard curve for *β*-carotene.

Otherwise, individual carotenoids, such as lycopene, have been determined in waste extracts from industrial tomato processing using HPLC, using a Hypersil ODS column (250 × 4.6 mm, 5 μm) with a mobile phase consisting of methanol–tetrahydrofuran–water 67:27:6 (*v*/*v*) in isocratic mode and a DAD at 472 nm [14]. Carotenoids have also been separated with a C30 RP column (250 × 4.6 mm) using gradient elution with a mobile phase consisting of methanol–methyl *tert*-butyl ether–water (81:17:2, *v*/*v*/*v*; eluent A) and methanol–methyl *tert*-butyl ether–water (10:88:2, *v*/*v*/*v*; eluent B) using a DAD at 450 nm. Under these conditions, lutein, *β*-cryptoxanthin, *α*-carotene, and 13-*cis*-*β*-carotene were tentatively identified [46]. Additionally, Hussain et al. determined β-carotene using HPLC with a DAD using a Carbon-30 column (4.6 mm i.d.) and a mixture of methanol–tertiary butyl methyl ether 80:20 (*v*/*v*) as the mobile phase [65]; lutein was quantitated from Citrus reticulata (kinnow) peel extracts using a combination of HPLC and UV–Vis with isocratic elution on a C18 column (150 × 4.6 mm, 5 μm) with a mobile phase consisting of MeOH, can, and triethylamine with a ratio of 900:100:1. The flow rate, injection volume, and detection wavelength were fixed at 1.0 mL·min^−1^, 25 μL, and 450 nm, respectively, along with a column temperature of 35 °C [111].

These results indicate that the total carotenoid content in agri-food waste extracts varies depending on the agri-food residue source. Spectrophotometric methods showed significant levels of carotenoids in tomato, pineapple, carrot, avocado, bael fruit, and pumpkin waste. A HPLC analysis identified specific carotenoids like lycopene, α- and β-carotene, and lutein in these extracts, with variations in the concentration and composition across different food wastes. These findings highlight the potential of agri-food waste as a valuable source of carotenoids for various applications.

### 5.4. Determination of Fatty Acids

The methods for the determination of fatty acids involve the use of GC with a previous derivatization step and temperature program. Different reagents have been employed to obtain derivatized fatty acids, including N-methyl-N-trimethylsilyltrifluoroacetamide [59], sodium methoxide [3,60], BF_3_ [19], KOH [61], NaOH [83], methanolic hydrogen chloride [62], and trimethylsulfonium hydroxide [91]. A wide range of columns have been used for the determination of fatty acid methyl esters (FAMES), such as DB-WAXUI (30 m × 0.25 mm i.d. × 0.25 μm d.f.) [3], DB-23 (60 m × 0.25 mm i.d. × 0.25 μm d.f.) [60], CP-SIL88 (100 m × 0.25 mm i.d. × 0.2 μm d.f.) [91], and VF-WAXms (60 m × 0.25 mm i.d.) [61] columns, as well as 50% cyanopropyl-methyl-50% phenylmethylpolysiloxane (30 m × 0.32 mm i.d. × 0.25 μm d.f.) [6] and poly(dimethyl)siloxane (30 m × 0.32 mm i.d. × 0.25 mm d.f.) [62] capillary columns.

Regarding GC detection, FAMES from agri-food waste samples have been frequently determined using MS [3,62] and flame ionization detection [6,19,60,61,91].

Different fatty acids have been identified in extracts from agri-food residues (Table 3). The most abundant determined have been oleic and linoleic acids in soybean residues (okara) [19], plums [3,83], and peach, apricot, and cherry seed oils [60]. In addition to oleic and linoleic acids, palmitic acid has been extracted in high amounts from chamomile waste [62], as have palmitic and α-eleostearic acids from sour cherry kernel oil [91]. Palmitic and stearic acids have been the most abundant in bio-residues from eggplant fruit [6], while cocoa waste flours have presented high contents of palmitic, stearic, oleic, linoleic, and linolenic acids [61].

Based on the fatty acid profile, some authors have also calculated the lipid nutritional quality indexes to estimate the nutritional values and possible health benefits of the obtained waste extracts, in terms of the total content of SFA, unsaturated fatty acids, MUFA, and PUFA; the atherogenicity, thrombogenicity, and desirable fatty acid index; and the hypocholesterolemic–hypercholesterolemic ratio [3,60].

In summary, bioactive fatty acids, such as oleic and linoleic acids, are abundant in soybean residues, plums, and various seed oils. Palmitic acid is prevalent in chamomile waste, while cocoa waste flours contain high levels of palmitic, stearic, oleic, linoleic, and linolenic acids. Nutritional quality indexes, including atherogenicity and thrombogenicity, have been calculated as essential parameters to assess the health benefits of these agri-food bio-residue extracts.

## 6. Evaluation of Bioactive Properties of Polyphenols, Tocopherols, Carotenoids, and Fatty Acids

The exploration of antioxidant, antibacterial, antifungal, anticancer, neuroprotective, and anti-inflammatory activities of bioactive compounds extracted from these bio-residues not only contributes to waste valorization but also opens new avenues for the development of functional foods and nutraceuticals. In this section, methods for evaluating the bioactive effects of extracts derived from agri-food biowaste will be explored in more detail, focusing on the potential health benefits of these extracts.

### 6.1. Methods for Antioxidant Activity Assessment

The evaluation of antioxidant activity in extracts derived from agri-food bio-residues is crucial due to their potential health benefits and applications in disease prevention. These bio-residues, often considered waste, are rich sources of bioactive compounds such as polyphenols, tocopherols, carotenoids, and fatty acids. These compounds exhibit significant antioxidant properties that can mitigate oxidative stress and contribute to the prevention of various diseases.

Polyphenols are known for their ability to scavenge free radicals and chelate metal ions, thereby inhibiting oxidative processes that can lead to chronic diseases such as cardiovascular diseases, cancers, and neurodegenerative disorders [4]. Tocopherols protect cellular membranes from lipid peroxidation, which is implicated in the pathogenesis of atherosclerosis and other inflammatory conditions [34]. Carotenoids, pigments found in plants, also contribute to antioxidant defense by quenching singlet oxygen and neutralizing free radicals, thus playing a role in reducing the risk of certain cancers and eye diseases [2]. Fatty acids, particularly unsaturated ones, have been shown to modulate oxidative stress and inflammation, which are key factors in the development of metabolic syndromes and other chronic conditions [104].

Measuring the antioxidant activity of these extracts is essential for several reasons. Firstly, it allows for the identification of bioactive compounds that can be utilized in the development of functional foods and nutraceuticals aimed at disease prevention. Secondly, it provides insights into the potential of these extracts to enhance human health by reducing oxidative damage and inflammation. Lastly, understanding the antioxidant capacity of these bio-residues supports the development of sustainable practices by valorizing agricultural waste, thereby contributing to environmental conservation and economic efficiency.

Several methodologies are often employed to assess the antioxidant properties of agri-food bio-residue extracts. Half of the research papers, in which methods for determining the antioxidant properties of extracts are used, employed two different methods [16], while only 26% used a single method [117], 21% used three methods [110], and 3% employed up to four different methods for determining antioxidant capacity [18]. The most commonly used method for antioxidant determination is the 2,2-diphenyl-1-picrylhydrazyl (DPPH) radical scavenging assay, representing 68% of the total articles measuring antioxidant capacity [118], followed by the 2,2′-azino-bis(3-ethylbenzothiazoline-6-sulfonic acid) (ABTS) radical scavenging assay (50%) [101], the ferric reducing antioxidant power (FRAP) method (47%) [106], and the thiobarbituric acid reactive substances (TBARS) assay (12%) [3].

The DPPH assay is a widely utilized method for evaluating the antioxidant activity of various compounds, including extracts from biological sources. This method is based on the reduction of the DPPH radical, a stable free radical characterized by a deep violet color with an absorption maximum of 517 nm. When an antioxidant compound donates a hydrogen atom to the DPPH radical, it reduces the DPPH to its non-radical form, resulting in a color change from violet to yellow. The degree of discoloration indicates the scavenging potential of the antioxidant compound or extract, which can be quantified by measuring the decrease in absorbance at 517 nm using UV–visible spectroscopy. The DPPH assay is favored for its simplicity, rapidity, and reproducibility, making it a standard method for the preliminary screening of antioxidant capacity in various samples.

DPPH assay results are typically calculated as the IC_50_ value, which is the concentration of the sample required to inhibit 50% of the DPPH radical activity. A lower IC_50_ value indicates higher antioxidant activity. However, to enable a comparison of the results between different studies, the outcome is typically expressed as TE. Among all the research papers that employed the DPPH method, only 57% used Trolox as a reference [19], 4% used gallic acid as a reference [119], and 39% did not use any reference [106], making it impossible to compare antioxidant capacity across different studies.

The ABTS assay is based on the ability of antioxidants to quench the ABTS radical cation, a stable blue-green chromophore with characteristic absorption at 734 nm. When an antioxidant compound donates an electron or hydrogen atom to the ABTS radical cation, it reduces the radical to a colorless form, resulting in a decrease in absorbance. The extent of decolorization is proportional to the antioxidant concentration and its scavenging ability. The ABTS assay is favored for its simplicity, sensitivity, and ability to measure both hydrophilic and lipophilic antioxidants, making it a versatile tool.

The ABTS assay results are typically expressed as TE, which standardizes the antioxidant capacity of the sample against a known antioxidant. This allows for the comparison of antioxidant activity across different studies. Among all the research papers that employ this method to determine antioxidant capacity, 94% used Trolox as a reference [36], making this method a good tool for comparison between different studies.

The FRAP assay is based on the reduction of ferric (Fe^3+^) to ferrous (Fe^2+^) ions in the presence of antioxidant compounds, which results in the formation of a blue-colored ferrous–tripyridyltriazine complex with an absorption maximum at 593 nm. The reduction reaction occurs in an acidic medium, where the antioxidants donate an electron to the ferric–tripyridyltriazine (Fe^3+^-TPTZ) complex, forming the ferrous–tripyridyltriazine (Fe^2+^-TPTZ) complex. The intensity of the blue color produced is directly proportional to the antioxidant compound concentration and its reducing power. The results are typically expressed as TE or Fe^2+^ equivalents. Among the research papers that employed the FRAP method, 44% used Trolox as a reference [104], 31% expressed it as iron equivalents [110], 6% expressed it as ascorbic acid equivalents [11], and 19% did not use any reference compound [62], making it impossible to make objective comparisons of these results.

The TBARS assay is a widely used method for evaluating lipid peroxidation, which is a key indicator of oxidative stress in biological systems. This method quantifies malondialdehyde (MDA), a secondary product of lipid oxidation, through a colorimetric reaction with thiobarbituric acid (TBA) under acidic conditions. The intensity at 532 nm of the previously formed pink chromogen, measured using UV–Vis spectroscopy, is directly proportional to the concentration of MDA, and thus, to the extent of lipid peroxidation. Despite its widespread use, the TBARS assay has some limitations, including potential interference from other substances that react with TBA. However, it remains a valuable tool for assessing oxidative stress and the antioxidant capacity of various compounds.

Among the research papers that employed the TBARS method, only 50% used Trolox as a reference [5], while the remaining papers provided antioxidant capacity results that are very difficult to compare [6].

Other less commonly used methods for determining antioxidant capacity, according to the research papers under study, include the oxygen radical absorbance capacity (ORAC) assay (9%) [2], cupric reducing antioxidant capacity (CUPRAC) assay (9%) [120], β-carotene/linoleic acid bleaching assay (6%) [18], phosphomolybdenum assay (3%) [109], the QUENCHER method (3%) [35], the dichloro-dihydro-fluorescein diacetate assay (3%) [5], and cell viability counting using Trypan Blue (3%) [106].

### 6.2. Methods for Antimicrobial Activity Assessment

The evaluation of antimicrobial properties, which encompasses both antibacterial and antifungal activities, is a critical aspect when investigating agri-food bio-residues. Identifying extracts rich in bioactive compounds with significant antimicrobial properties can lead to the development of new antibiotics and antifungal agents, which is particularly important in the context of rising antimicrobial resistance that poses a significant threat to global health. Assessing their efficacy is vital for controlling and preventing infections caused by pathogenic bacteria and fungi. This includes applications in human and veterinary medicine, as well as in agriculture to protect crops from microbial diseases.

Additionally, the antimicrobial activity of bioactive compounds is crucial in the food industry to prevent microbial contamination and extend the shelf life of food products, ensuring food safety and reducing food waste. Measuring these activities provides valuable insights into the mechanisms of action of various bioactive compounds, driving innovation in biotechnology and the development of new applications for extracts rich in these compounds. This not only contributes to the development of new therapeutic agents but also enhances food safety and supports sustainable agricultural practices.

Among the Gram-positive bacteria most commonly employed to assess the antibacterial activity of extracts with bioactive properties are *Staphylococcus aureus* (*S. aureus*) [10], *Bacillus cereus* (*B. cereus*) [36], and *Listeria monocytogenes* (*L. monocytogenes*) [105]. Gram-negative bacteria employed in these studies include *Escherichia coli* (*E. coli*) [6], *Pseudomonas aeruginosa* (*P. aeruginosa*) [5], and *Salmonella enterica* (*S. enterica*) [102]. Assessment studies of antifungal activity often include the strain *Aspergillus fumigatus* (*A. fumigatus*) [6], the most studied fungal strain in bioactive extracts when assessing this parameter due to its high pathogenicity.

The Kirby–Bauer test, also known as the disk diffusion method, is a widely used technique to assess antibacterial activity. This method involves placing paper disks impregnated with specific concentrations of bioactive extracts onto the surface of an agar plate previously inoculated with the bacteria of interest. As the bioactive compounds of the extract diffuse from the disk into the agar, they create a gradient of concentration. Bacteria susceptible to the bioactive extract will be inhibited from growing around the disk, forming a clear zone known as the zone of inhibition [98]. The size of the inhibition zone indicates the bacteria’s sensitivity to the extract. Larger zones suggest greater susceptibility, while smaller zones indicate resistance. Some authors state that extracts that present an inhibition halo of more than 7 mm can be considered potential antibacterial [105]. However, this method only provides a qualitative result.

The most common parameter that quantitatively indicates the antimicrobial activity of a bioactive extract is the minimum inhibitory concentration (MIC), and is the lowest concentration of an antimicrobial agent that inhibits the visible in vitro growth of microorganisms. Methods such as broth microdilution or agar microdilution are commonly used to determine the MIC value [36]. This value is crucial for determining the appropriate dosage of bioactive compounds for treating infections.

Another important parameter is the minimum bactericidal (MBC) [5] or fungicidal (MFC) [10] concentration, being the lowest concentration of an antimicrobial agent required to kill a particular bacterium or fungus. The MBC–MFC can be determined after the MIC evaluation by subculturing the contents of the tubes or wells that showed no visible growth onto fresh agar plates. The plates are then incubated, and the MBC–MFC are identified as the lowest concentration at which no bacterial or fungal colonies grow, indicating that the microbials have been killed. Generally, if the MBC is less than four times the MIC, antibacterial extracts can also be considered bactericidal [121]. The MBC–MFC provides valuable information about the bactericidal or fungicidal efficacy of an antimicrobial agent, or, on the other hand, its bacteriostatic or fungistatic effect if they are only able to inhibit their growth.

### 6.3. Methods for Anticarcinogenic, Neuroprotective, and Anti-Inflammatory Activity Assessment

Determining the anticarcinogenic activity of bioactive extracts aids in the identification and development of potential therapeutic formulations that can prevent or treat some kinds of cancer. By understanding the mechanisms through which these compounds exert their effects, it is possible to design more effective and targeted treatments, ultimately contributing to the reduction of cancer incidence and mortality. The integration of these methodologies provides a robust framework for the systematic evaluation of anticarcinogenic properties, thereby advancing the development of potential therapeutic agents. Frequently tested human tumor lines include colon cancer (Caco-2), cervical carcinoma (HeLa), hepatocellular carcinoma (HepG2), breast cancer (MCF-7), and lung cancer (NCI-H460) [5,6].

Among the most commonly used methods to determine the cytotoxicity of extracts rich in bioactive compounds against tumor cell lines are the 3-(4,5-dimethylthiazol-2-yl)-2,5-diphenyltetrazolium bromide (MTT) and the sulforhodamine B (SRB) colorimetric assays. The MTT assay measures cellular metabolic activity as an indicator of cell viability, while the SRB assay quantifies cellular protein content to assess cell density and growth inhibition. Together, these methods offer a comprehensive approach to understanding the cytotoxic effects of bioactive extracts on cancer cells. The SRB assay relies on the ability of sulforhodamine B, a bright-pink aminoxanthene dye that absorbs at 540 nm, to bind to cellular protein components under mildly acidic conditions. The amount of dye bound to the proteins is proportional to the total protein content of the cells, serving as an indirect measure of cell density. The SRB assay is particularly useful for determining cell growth inhibition, cytotoxicity, and drug sensitivity [5]. Its advantages include simplicity, reproducibility, and the ability to provide quantitative data on cell proliferation and cytotoxicity. Data are usually reported as the extract concentration required to inhibit 50% of the cell growth (GI_50_) [6].

The assessment of neuroprotective activity involves various methodologies to evaluate the efficacy of bioactive extracts in protecting neuronal cells from damage and degeneration. The aggregation of amyloid-beta (Aβ_42_) peptides is a hallmark of Alzheimer’s disease, leading to the formation of toxic oligomers and fibrils that contribute to neuronal damage. Transmission electron microscopy (TEM) is frequently employed to visualize and quantify the extent of amyloid fibril aggregation. TEM provides high-resolution images that allow us to observe the morphology of amyloid fibrils and assess the effectiveness of aggregation inhibitors [3].

Acetylcholinesterase (AChE) and butyrylcholinesterase (BChE) are enzymes involved in the breakdown of acetylcholine and butyrylcholine, essential neurotransmitters for cognitive function. Inhibitors of AChE and BChE are used to increase choline levels in the brain, thereby enhancing cholinergic transmission and providing neuroprotective effects. The activity of these enzymes can be measured using fluorescence spectroscopy, which quantifies the hydrolysis of specific substrates of AChE and BChE [2].

The anti-inflammatory properties of extracts can be evaluated by means of the method of lipopolysaccharide (LPS)-induced nitric oxide production employing various macrophage cell lines. These cells are treated with LPS to induce an inflammatory response, catalyzing the production of nitric oxide from L-arginine, being converted into nitrite. Nitrite is then quantified colorimetrically by the reaction with Griess reagents to form a pink azo dye that absorbs between 540 and 570 nm. With the co-treatment of cells employing various concentrations of bioactive extracts, their anti-inflammatory activity can be quantified [5,6].

### 6.4. Bioactive Properties of Agri-Food Bio-Residue Extracts

The following section describes the most important bioactive properties of extracts from agri-food bio-residues as reported in the literature, as well as the methods used to evaluate these properties (Table 4).

Ferreira et al. evaluated the antioxidant and neuroprotective properties of the methanolic extract of pear pomace flours from *Pyrus communis* L., var. Rocha, rich in flavonoids, tocopherols, carotenoids, and fatty acids [2]. The antioxidant activity measured using the ABTS method yielded a result of 2.3–3.0, while the ORAC method provided 5.3–6.1 mmol TE·100 g^−1^ of dry weight. Regarding neuroprotection, the percentage of AChE inhibition at 100 mg·mL^−1^ of extract was 15.10–23.43%, far from the positive control physostigmine salicylate (61.67%), similar to BChE (9.61–16.04% vs. 57.27%). These antioxidant and neuroprotective results are modest, but they reveal the bioactive role of the compounds present in the extracts of pear pomace flours.

Rodríguez-Blázquez et al. evaluated the antioxidant, antibacterial, and neuroprotective properties of oil extracts, rich in unsaturated fatty acids and tocopherols, and defatted ethanol–water extracts, rich in neochlorogenic acid, chlorogenic acid, and protocatechuate, from plum (*Prunus domestica* L.) seeds [3]. The antioxidant activity measured using the DPPH method yielded an IC_50_ of 10–36 mg·mL^−1^ for oil extracts, while 0.9–1.9 mg·g^−1^ was obtained for defatted phenolic extracts. Employing the TBARS method, an IC_50_ of 1.3–5.0 mg·g^−1^ was obtained for phenolic extracts—slightly worse results than those obtained using the DPPH method. Regarding antibacterial properties, phenolic extracts showed MIC values equal to or higher than 20 mg·mL^−1^ for both *E. coli* and *S. aureus* strains. A neuroprotective assay, expressed as the inhibition of Aβ aggregation visualized by TEM, showed the ability of plum seed phenolic extract to totally inhibit fibril formation at 25 mg·mL^−1^ of extract concentration. This extract was also able to reduce fibril width when compared to the control (30%).

Albuquerque et al. studied the antioxidant, antimicrobial, anticarcinogenic, and anti-inflammatory activities of the hydroethanolic extracts of the seeds and peels of Brazilian berry grumixama (*Eugenia brasiliensis* Lam.) waste [5]. Entire residue extracts were rich in tocopherols and flavonoids while seed extracts possessed a higher content of MUFA and peel extracts had a mixed profile between them. Half maximal effective concentration (EC_50_) values of 0.90–1.34 µg·mL^−1^ were obtained using the TBARS method, five times lower than Trolox (5.8 µg·mL^−1^). The antibacterial activity of the entire residue, seed, and peel extracts against Gram-positive bacteria (*S. aureus*, *B. cereus*, *L. monocytogenes*) offered MICs of 0.3125–2.5 mg·mL^−1^, 0.156–2.5 mg·mL^−1^, and 0.078–0.625 mg·mL^−1^, respectively. Against Gram-negative bacteria (*E. coli*, *P. aeruginosa*, *S. enterica*, and *Y. enterocolitica*), MIC values were 0.078–5 mg·mL^−1^, 0.156–2.5 mg·mL^−1^, and 0.625–20 mg·mL^−1^, respectively. On the other hand, the lowest MBC was found for *Y. enterocolitica* (0.3125–2.5 mg·mL^−1^). Antifungal activity (*A. fumigatus* and *A. brasiliensis*) was demonstrated by the obtention of MICs of 0.3125–1.25 mg·mL^−1^, with insufficient values of MFC (>20 mg·mL^−1^). Therefore, they could only be considered bactericidal, not fungistatic, extracts. The seed extract presented the best anti-inflammatory activity using the LPS-NO method (EC_50_ = 98 µg·mL^−1^), with the peel extract presenting the worst result (EC_50_ > 400 µg·mL^−1^). Anticarcinogenic activity, tested against human tumor cell lines (AGS, Caco-2, MCF-7, and NCI-H460) with a SRB assay, was again better for seed extracts (GI_50_ = 14.7–62 µg·mL^−1^) than for peel extracts (GI_50_ = 67–186 µg·mL^−1^). The authors stated that further studies are necessary to unveil the mechanisms of these biological properties.

Silva et al. evaluated the antioxidant, antimicrobial, anti-inflammatory, and anticarcinogenic activities of eggplant fruit (*Solanun melongena* L.) residues, rich in unsaturated fatty acids and polyphenols [6]. The authors compared extracts from different fruit parts: whole fruit, peel, and pulp. Epicarp extract showed the best antioxidant activity (EC_50_ = 135 µg·mL^−1^), forty times more effective than the pulp extract (EC_50_ = 4300 µg·mL^−1^), employing the TBARS method. However, the authors did not use any reference compound. Antibacterial activity against Gram-negative (*E. cloacae*, *E. coli*, and *S. Typhimurium*) and Gram-positive (*L. monocytogenes* and *S. aureus*) bacteria was almost similar for all extracts (MIC = 2–8 mg·mL^−1^; MBC = 4–8 mg·mL^−1^). These extracts showed almost no antifungal activity against *A. fumigatus*, *A. niger*, *A. versicolor*, *P. funiculosum*, and *T. viride* (MIC and MFC > 8 mg·mL^−1^), but pulp extract was effective against *P. ochrochloron*, inhibiting the growth (MIC = 1 mg·mL^−1^) and also being fungicidal (MFC = 1 mg·mL^−1^). None of the pulp or whole fruit extracts, at the concentrations evaluated, possessed anticarcinogenic activity against the human tumor cell lines tested (HeLA, HepG2, MCF-7, and NCI-H460) with a SRB assay (GI_50_ > 400 µg·mL^−1^). However, epicarp extracts’ anticarcinogenic activity was observed against HepG2 (GI_50_ = 280 µg·mL^−1^), HeLa (GI_50_ = 337 µg·mL^−1^), and NCI-H460 (GI_50_ = 340 µg·mL^−1^). Data for anti-inflammatory activity measured using the LPS-NO method were not reported.

Rodrigues et al. tested the antioxidant and antimicrobial properties of green extracts (hydroethanolic and aqueous) of chestnut (*Castanea sativa* Mill.) burs, shells, and leaves, which were rich in phenolic compounds [10]. Antioxidant activities were measured using three different methods: DPPH, TBARS, and FRAP. By means of the DPPH method, the extracts of leaves (EC_50_ = 0.18–0.27 mg·mL^−1^), shells (EC_50_ = 0.07–0.90 mg·mL^−1^), and burs (EC_50_ = 0.14–0.33 mg·mL^−1^) presented lower antioxidant capacity than trolox (EC_50_ = 0.043 mg·mL^−1^). While TBARS values of leaf (EC_50_ = 0.08–2.0 mg·mL^−1^) and shell (EC_50_ = 0.018–0.56 mg·mL^−1^) extracts were also worse than those of Trolox (EC_50_ = 0.0058 mg·mL^−1^), bur extracts (EC_50_ = 0.002–0.008 mg·mL^−1^) demonstrated higher antioxidant capacity than the former using this method. Regarding the FRAP assay results, none of the extracts (EC_50_ = 0.07–1.34 mg·mL^−1^) resulted better than Trolox (EC_50_ = 0.029 mg·mL^−1^). Taking these results into account, bur extracts presented the highest antioxidant capacity using the TBARS method, overcoming Trolox, while shell extracts presented the best antioxidant capacity using DPPH and FRAP assays, but were lower than Trolox. Antibacterial activity was evaluated against Gram-negative (*E. cloacae*, *E. coli*, *P. aeruginosa*, *S. enterica*, and *Y. enterocolitica*) and Gram-positive (*B. cereus*, *L. monocytogenes*, and *S. aureus*) bacteria. Bur extracts were the most bactericidal, with MBC values of 5 mg·mL^−1^, and were the only extracts able to inhibit the growth of *E. coli* at a concentration lower than 10 mg·mL^−1^. Leaf extracts were able to inhibit the growth of all bacteria tested, with MIC values equal to or lower than 10 mg·mL^−1^, being therefore bacteriostatic extracts. Regarding antifungal activity against *A. fumigatus* and *A. brasiliensis*, no extract demonstrated fungicidal properties, bur extracts being the only ones with fungistatic activity (MIC = 10 mg·mL^−1^). The authors demonstrated that the solvent, and the extraction method employed, influenced both the antioxidant and antimicrobial activities of chestnut extracts.

Romero et al. studied the antioxidant and antifungal activities of quince (*Cydonia oblonga* Mill.) by-products, which were rich in polyphenols [122]. Phenolic extracts presented 72% of DPPH inhibition, although a comparison with a reference compound was not carried out. Using the ABTS method, extracts showed a value of 380 µmol TE·100 g^−1^ of extract. Antifungal activity measured using the disk diffusion method against two phytopathogenic fungi (*B. cinerea* and *C. acutatum*) did not offer promising results since it was not able to inhibit the growth of any of them at the tested concentrations.

Gaharwar et al. evaluated the antioxidant and antibacterial properties of pomegranate (*Punica granatum*) peel extracts, rich in phenolic compounds, using green DESs [98]. By means of the ABTS assay, pomegranate peel extracts presented values of 15–30 mM TE, while with the FRAP assay, the values were 45–100 mmol of iron equivalents per L. The antibacterial activity against Gram-positive (*S. aureus*) and Gram-negative (*E. coli*) bacteria was evaluated using the agar diffusion method. These extracts showed greater inhibition against *S. aureus* than against *E.coli*. However, the inhibition zones were moderate (<4 mm).

Dauber et al. investigated the antioxidant and antibacterial potential of alperujo residue from the olive oil industry of Arbequina and Coratina cultivars, which were rich in phenolic compounds and tocopherols [36]. In general, these extracts presented ABTS values of 0.607–1.224 mg TE·g^−1^, while the ORAC values were 180–318 mg TE·g^−1^. Antibacterial activity tested against Gram-negative (*E. coli*, *S. typhimurium*, and *K. pneumoniae*) and Gram-positive (*S. aureus*, *B. subtilis*, and *B. cereus*) bacteria offered MIC values of 1.3 mg·mL^−1^ for Coratina extracts and 0.6–1.3 mg·mL^−1^ for Arbequina extracts for most of the bacteria. However, Coratina extracts could not inhibit *E.coli*, and Arbequina extracts could not inhibit *E.coli*, *S. typhimurium*, or *B. subtilis*. Together, these results showed the higher antioxidant and antibacterial of Coratina alperujo extracts versus Arbequina ones.

Massironi et al. employed extracts from medicinal plant saw palmetto (*Serenoa repens*) residues, with a high content of fatty acids and phenolic compounds, to test their antioxidant and antibacterial activities [123]. Extracts obtained at mild temperature (150 °C) presented higher antioxidant values using ABTS (140 mg TE·g^−1^ dry extract) and DPPH (0.088 mg TE·g^−1^) assays, in contrast to extracts obtained at 120 °C (76 mg and 0.031 mg TE·g^−1^, respectively) and 180 °C (39 mg and 0.036 mg TE·g^−1^, respectively). Antibacterial activity was tested against Gram-negative (*E. coli*) and Gram-positive (*L. innocua* and *B. cereus*) bacteria employing the broth dilution method. The MBC values for the extract obtained at higher temperatures were 12.5–100 mg·mL^−1^, and it was the only extract that was bactericidal against *E. coli*. However, MBC values against Gram-positive bacteria for extracts obtained at 120 °C and 150 °C were 15–100 mg·mL^−1^. The authors stated that the saw palmetto residue extract obtained at higher temperatures can be considered the most effective.

Santos et al. evaluated the antioxidant and antibacterial potential of acerola cherry (*Malpighia emarginata*) residue, rich in several polyphenols [105]. Using the DPPH method, acerola residue extract presented 3.5 g TE·100 g^−1^ of dry sample, therefore being highly antioxidant. Antibacterial activity was tested using the disk diffusion method against *S*. *aureus*, *C*. *perfringens*, *S*. *Typhimurium*, *B*. *cereus*, *L*. *monocytogenes*, *L*. *innocua*, and *E*. *coli* [105]. Acerola extract showed inhibition against *S*. *Typhimurium* at a concentration of 25% (halo of 8.2 mm) and against *L*. *monocytogenes* at a concentration of 100% of the extract (halo of 7.9 mm). However, the authors did not express this concentration per gram of extract, which makes it difficult to compare with other studies.

Bouloumpasi et al. assessed the antioxidant and antibacterial potential of phenolic extracts from solid residues of oregano (*Origanum vulgare*), rosemary (*Rosmarinus officinalis*), sage (*Salvia fruticosa*), lemon balm (*Melissa officinalis*), and spearmint (*Mentha spicata*) [102]. Antioxidant activity was measured using DPPH, ABTS, and FRAP assays, offering slightly similar results within the same extract. Lemon balm extract was the most antioxidant extract (500–700 mg GAE·g^−1^), followed by spearmint (350–500 mg GAE·g^−1^), oregano (300–450 mg GAE·g^−1^), sage (300–350 mg GAE·g^−1^), and rosemary (250–300 mg GAE·g^−1^). Antibacterial activity was measured using a broth microdilution assay at four different extract concentrations (0.5, 0.75, 1.5, and 3.0 g·L^−1^) against *E. coli*, *S. aureus*, *B. subtilis*, *B. licheniformis*, *S. Typhimurium*, *L. monocytogenes*, *B. licheniformis*, and *B. cereus* [102]. Some of the Gram-positive bacteria (*S. aureus*, *L. monocytogenes*, *B. subtilis*, and *B. licheniformis*) were the most sensitive, and some of the Gram-negative bacteria (*S. Typhimurium* and *E. coli*) were the most resistant. Rosemary, sage, and spearmint extracts showed the highest inhibition overall to most of the bacteria tested (up to 100% inhibition). Oregano and lemon balm were not as effective, with bacterial growth inhibitions up to 20%.

As demonstrated, the presence of polyphenols, tocopherols, carotenoids, and fatty acids in agri-food bio-residues provides multiple health benefits. These bioactive compounds are known for their diverse and potent properties, which include antioxidant, antibacterial, antifungal, anticancer, neuroprotective, and anti-inflammatory activities. The synergistic effects of these compounds contribute significantly to the overall bioactivity of agri-food bio-residues, making them valuable resources for developing health-promoting products.

## 7. Potential Applications of Bioactive Compounds

Owing to their intrinsic qualities and added value, encompassing antioxidant, anti-inflammatory, antimicrobial, anticancer, and neuroprotective activities among others, bioactive compounds derived from vegetable agri-food waste have emerged as compelling and cost-effective functional ingredients across various industrial sectors. These bioactives facilitate the development of clean-label, functional products that pose fewer risks to both the environment and human health. Furthermore, this aligns with the prevailing consumer trend towards vegan and synthetic compound-free products [21,22,68].

**Figure 5:**
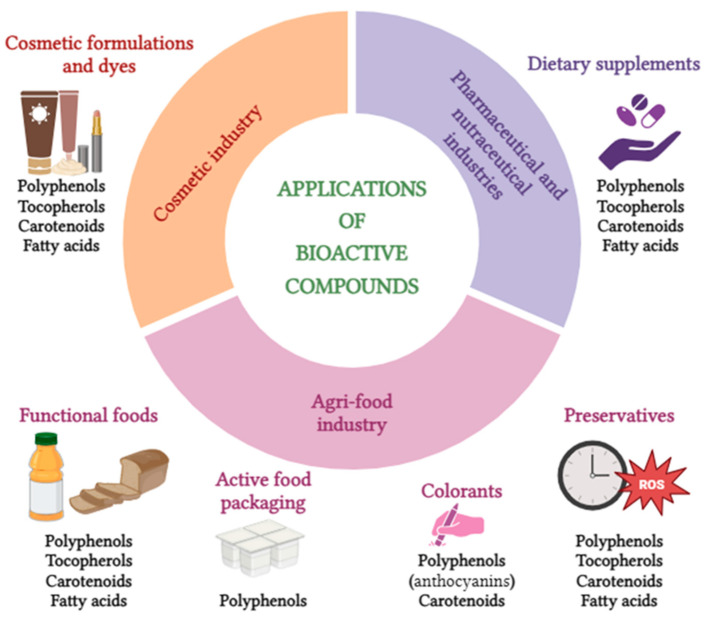
Established and innovative industrial applications of polyphenols, carotenoids, tocopherols, and fatty acids.

The following lines describe some of the possible uses and applications of the bioactives analyzed in this review, which are also described graphically in Figure 5.

The pharmaceutical industry is one of the most obvious and direct uses of polyphenols, tocopherols, and carotenoids; as a result, these substances can be used for both preventing and treating carcinoma, heart conditions, and neurological conditions, to mention a few. Additionally, polyphenols and tocopherols have proven effective in addressing the current issue of microbial multidrug resistance, serving as valuable and efficient antibiotics and antifungals [105,124]. A notable example is astaxanthin, a carotenoid with potent antioxidant activity that can cross the blood–brain barrier, conferring significant neuroprotective properties. Furthermore, it has been shown to inhibit the expression of matrix metalloproteinases and prevent lipid peroxidation, making it useful in the treatment of cancer and inflammatory diseases [125]. Furthermore, elevated levels of α-tocopherol are associated with a reduced risk of cognitive impairment in older adults, modulated by concurrent cholesterol concentration [124]. Additionally, acerola cherry extracts, rich in gentisic and protocatechuic acids, inhibit the growth of *Salmonella typhimurium* [105], and bioactive extracts rich in anthocyanins (cyanidin and peonidin derivatives) and flavonoids such as myricetin and quercetin derivatives have demonstrated the ability to inhibit inflammation, multiplicity, and the incidence of colon cancer in vivo [22].

In terms of the agri-food industry, polyphenols, carotenoids, tocopherols, and fatty acids could be applied as ingredients in the enhancement of functional foods, i.e., foods that promote physical health and lower the risk of specific illnesses. Additionally, based on their antioxidant, antimicrobial, and UV protection properties, their use as natural food preservatives has been promoted, as they can increase the shelf life and sensory quality of foods [21,22]. For instance, phenolic extracts derived from the processing shells of Camellia oleifera effectively inhibited Listeria monocytogene contamination, prevented lipid oxidation, and delayed the migration of moisture in sea bass fillets for a duration of one week [126]. Moreover, oil obtained from plum seeds, rich in tocopherols and PUFA, as well as with notable antioxidant activity, has been proposed for the design of functional foods [3].

Furthermore, bioactive compounds can be employed as natural colorants, highlighting their greater biocompatibility, biodegradability, non-toxicity, and non-allergenic effects compared to synthetic colorants. In this field, the applicability of anthocyanins stands out, as their color changes with pH. As such, they have been used in the design of macarons, soft drinks, yogurt, and syrup, showing an attractive and stable color and high sensory acceptance [21,22]. Additionally, carotenoids from mandarin peels and tomatoes have been used to develop bakery and yogurt products with intense yellow and red colors, respectively [22,125]. Similarly, natural bioactive compounds have been introduced into active and intelligent food packaging systems. Various studies have focused on developing smart packaging that indicates food freshness through the incorporation of anthocyanins and proanthocyanidins in polymers, acting as structural elements and pH and CO_2_ indicators [22].

Positioned at the intersection of the pharmaceutical and agri-food sectors, nutraceuticals represent concentrated sources of nutrients or bioactive compounds with both nutritional and therapeutic properties. These are generally classified as dietary supplements, and their market has witnessed exponential growth in recent years, including supplements such as carotenoids, vitamin E and α-tocopherol, omega-3 and omega-6 fatty acids, and polyphenols like resveratrol [21,22,41,124,125].

On the opposite end of the spectrum, the cosmetic industry has increasingly turned to natural bioactive compounds. This shift is driven by the rising consumer demand for natural products and the banning of certain synthetic compounds due to safety concerns [22]. Notably, stilbenes, flavonoids, and hydroxycinnamic acids are known for their exceptional UV absorption, making them ideal for sunscreen formulations [127]. Moreover, the antioxidant and anti-aging properties of bioactive compounds are being harnessed for the development of skincare creams. For example, *Euryale ferox* peel, containing complex tannins and flavanols, has demonstrated significant potential as an anti-tyrosinase agent and melanin production inhibitor [128]. Additionally, carotenoids and anthocyanins are being utilized as natural colorants, offering a safer alternative to synthetic dyes and reducing allergy risks [22]. Moreover, vegetable oils originating from bio-residues such as soybean and *Prunus* seeds function as highly effective emulsifiers in cosmetic formulations, with additional health benefits enhanced by their rich content of tocopherols and fatty acids [3,22]. Overall, this multifaceted approach not only meets consumer preferences but also enhances the safety and efficacy of cosmetic formulations.

## 8. Limitation in Agri-Food Bio-Residues Valorization: Potential Toxic Substances and Techno–Economic Considerations

The valorization of agri-food bio-residues for the extraction of bioactive compounds is a promising approach for sustainable development. However, the potential presence of toxic substances in these residues poses significant concerns for food safety and human health [129]. Food by-products can contain microorganisms such as *Salmonella*, *Listeria*, and *E. coli*, as well as chemical contaminants like pesticides, heavy metals (lead, cadmium, mercury), and mycotoxins (aflatoxins, ochratoxin A). Ensuring the safety of these by-products is crucial for their valorization, and specific regulations are needed to guarantee their safety [130].

Alkaloids are naturally occurring compounds found in various plants, often characterized by their nitrogen-containing heterocyclic structures. These compounds can exhibit high toxicity when present in food compounds. Tropane alkaloids (TAs) (such as atropine and scopolamine), found in plants like belladonna and jimson weed, are known for their anticholinergic effects, which can lead to the development of neurodegenerative diseases [131]. Pyrrolizidine alkaloids, present in plants like Senecio and Crotalaria, are hepatotoxic and genotoxic, posing significant health risks through liver damage and cancer [132]. Glycoalkaloids (such as solanine and chaconine), found in potatoes and tomatoes, can cause gastrointestinal and neurological symptoms, necessitating careful monitoring to prevent toxic exposure [133]. Alkaloids can be detected in food matrices, often resulting from the accidental inclusion of alkaloid-containing weeds during harvesting or processing [134]. Meanwhile, the contamination of soybeans with TAs, such as atropine and scopolamine, is very frequent [135,136], and there are no current regulations related to the maximum level allowed [135]. The presence of these toxic alkaloids in agri-food bio-residues necessitates the development of sensitive and reliable analytical methods for their detection and quantification. HPLC–MS/MS has been shown to be effective in the high-throughput determination of multiple toxic alkaloids in food matrices [137].

Amygdalin, a cyanogenic glycoside, is another toxic compound that can be present in agri-food bio-residues. Found in the seeds and kernels of fruits such as apricots, peaches, and plums, amygdalin can release hydrogen cyanide upon hydrolysis, posing a risk of cyanide poisoning [138]. The potential cytotoxic effects of amygdalin have been evaluated in various studies, highlighting the need for careful monitoring and regulation of its presence in food products [139].

Therefore, stringent regulatory frameworks and sustainable agricultural practices are essential to minimize the content of toxic compounds and ensure food safety. Future research should focus on developing more efficient and eco-friendly selective extraction methods that prevent the co-extraction of these natural substances or detoxification processes to exploit extracts rich in bioactive compounds without the disadvantages caused by toxic compounds.

Despite the promising potential of agri-food residues to enhance global energy supplies, as highlighted throughout this review, it is crucial to emphasize that the success of waste-to-wealth initiatives hinges on evaluating the economic feasibility of bio-residue beneficiation from both techno–economical and logistical perspectives. In recent years, numerous studies have focused on the improvement or valorization of agri-food residues, although less than a tenth include any form of techno–economic analysis, being crucial to delving into the potential value of the promoted value-added products or processes to estimate the feasibility of the processes developed at the laboratory scale [140,141].

One of the main obstacles is the variability in the composition of the residues, which complicates the standardization of conversion processes and affects the efficiency and quality of the final products. Additionally, the infrastructure required demands significant investments, which can be a barrier to large-scale implementation [141]. Furthermore, the capacity or size of the plant is one of the most influential parameters in the profitability of the valorization process, considering that bio-residues are often generated seasonally and degrade rapidly due to their high moisture and organic matter content. Therefore, the need for efficient collection and transportation of agri-food residues can increase operational costs and reduce the competitiveness of valorized products [140].

Moreover, the profitability of valorization projects is heavily impacted by market price fluctuations. Along these lines, it has been indicated that the process is stronger when multiple products are obtained [140], with studies based on the sequential or simultaneous extraction of different bioactives, such as the proposal by Rodríguez-Blázquez et al. [3] and Kanaan et al. [142], to obtain oils rich in tocopherols and/or fatty acids, followed by hydrophilic extracts rich in phenolic compounds. Although product depletion must be considered, it can be directed towards other biomass goods such as biochar, biofuel, or nanocellulose, among others [141]. Despite these limitations, continuous research and the development of supportive policies can help overcome these obstacles and promote the sustainable valorization of agri-food bio-residues, which offers a mutually beneficial solution, fostering both environmental sustainability and global economic growth.

## Figures and Tables

**Table 1:** Phenolic compounds, tocopherols, carotenoids, and fatty acids from primary agri-food waste sources.

Processed Food	Food Waste	Food Waste Produced (%)	Bioactive Compounds	Ref.
Apple	Pomace Peels	25–30	Phenolic acids (hydroxycinnamic aids) Flavonoids (flavonols, flavanols) Tannins Tocopherols	[22,48,58]
*Prunus* fruits (apricot, cherry, peach, plum)	Pomace Stone (shell and seeds)	30–60	Phenolic acids (hydroxybenzoic and hydroxycinnamic acids) Flavonoids (flavonols, flavones, flavan-3-ols, anthocyanins) Tannins Tocopherols Carotenoids Fatty acids	[35,67]
*Citrus* fruits (lemon, lime, orange, tangerine, etc.)	Peels Seeds Leaves	50	Phenolic acids (hydroxybenzoic and hydroxycinnamic acids) Flavonoids (flavonols, flavones, flavan-3-ols, anthocyanins) Tocopherols	[59,68]
Avocado	Peels Seeds Leaves	30	Phenolic acids (hydroxybenzoic and hydroxycinnamic acids) Flavonoids (flavonols, flavanones, flavanols, flavones) Lignans Tannins Fatty acids	[29,69]
Tomato	Branches Pomace Peels	8	Phenolic acids (hydroxy-cinnamic acids) Flavonoids Carotenoids Fatty acids	[22,40,63]
Soybean	Soybean meal; soybean whey Okara	Up to 90%	Flavonoids (isoflavones) Fatty acids	[70]
Wine	Vine leaves Vine shoots Grape stalks Grape pomace Grape seeds Wine lees	15–20	Phenolic acids (hydroxybenzoic acids) Flavonoids (flavonols, flavanols, anthocyanins) Stilbenes Tannins (condensed tannins) Fatty acids	[53]
Beer	Spent grain Trub Spent yeast Spent kieselguhr	15–85	Phenolic acids (hydroxybenzoic and hydroxycinnamic aids) Flavonoids (flavanones, flavanols) Tannins Fatty acids	[71]
Coffee	Coffee pulp Spent coffee grounds	50	Phenolic acids (hydroxybenzoic and hydroxycinnamic aids) Lignans	[22,72]

**Table 2:** TPC and major polyphenols or polyphenol families found in agri-food bio-residues.

Residues	TPC (mg GAE·g^−1^)	Total Subclass Content	Major Polyphenols or Families	Ref.
Plum seed (*Prunus domestica* L.)	0.57–2.40	TFC: 0.9–1.3 mg QE·g^−1^	Hydroxybenzoic acids (5) Hydroxycinnamic acids (5) Flavanols (7)	[3]
Acerola (*Malpighia emarginata*)	9	TAC: 0.57 g·g^−1^ Flavones: 3.3 g·g^−1^ Tannins: 0.35 mg TAE·g^−1^	Phenolic acids (10) Flavonoids (6)	[105]
Açaí (*Euterpe oleracea Mart.*) seeds, slurry, and pulp	28.6 (pulp) 17.7 (seeds) 1.6 (slurry)	TFC: 0.82–2.53 mg GAE·g^−1^ TAC: 2.93 mg·g^−1^ (pulp)	Not reported	[80]
Taro (*Colacasia esculenta* L.) leaves	5.0–24.0	TFC: 2.5–6.0 mg CE·g^−1^ TF: 4.0–8.0 mg QE·g^−1^ THA: 2.0–4.5 mg CAE·g^−1^	Flavones (8) Flavonols (3) Caffeic acid derivative (1)	[106]
Pomegranate peel (*Punica granatum*)	3.5–10 g GAE·L^−1^	Flavonoids and tannins (qualitative test)	Ellagic acid Gallic acid Ferulic acid p-Coumaric acid Protocatechuic acid Caffeic acid	[98]
Pomegranate peel (*Punica granatum*)	115–249	Not reported	Phenolic acids (2) Ellagitannins (8)	[16]
Peru cocoa (*Theobroma cacao*)	4.9–31.3	Flavan-3-ol: 19–130 mg CE·g^−1^	Flavonoids (29) Phenolic acids (5)	[27]
Brazilian berry (*Eugenia brasiliensis* Lam.) seeds, peels, and whole residue	118.5–134.0 mg·g^−1^	Hydrolyzable tannins: 13–104 mg·g^−1^ Condensed tannins: 27 mg·g^−1^ Anthocyanins: 12.6–77 mg·g^−1^	Hydrolyzable tannins (17) Condensed tannins (7) Anthocyanins (4)	[5]
Avocado (*Persea americana*) peel	31.3–40.6	TFC: 61.3–70.5 mg RE·g^−1^	Flavonoids (22) Phenolic acids (21) Lignans (2)	[29]
Pineapple (*Ananas comosus* L.) fruit and peel	14.4–54.8	TFC: 26.9–49.7 mg·g^−1^	Hydroxybenzoic acids Hydroxycinnamic acids	[109]

**Table 3:** Major tocopherols, carotenoids, and fatty acids found in agri-food bio-residues.

Residues	Bioactive Family	Target Compounds (mg·kg^−1^)	Instrumental Techniques	Ref.
Plum seed (*Prunus domestica* L.)	Tocopherols	α-tocopherol (2.0–2.5) β- and γ-tocopherol (5.7–11.2) δ-tocopherol (1.5–2.2)	HPLC–DAD	[3]
Alperujo *Arbequina* and *Coratina*	Tocopherols	α-tocopherol (173–335) β- and γ-tocopherol (27–95) δ-tocopherol (17–25)	HPLC–fluorescence	[36]
Pitted sour cherry (*Prunus cerasus* L.) pomace	Tocopherols	α-tocopherol (81.4–195.9) β- and γ-tocopherol (45.6–272.0) δ-tocopherol (11.4–27.9)	UHPLC–QTOF–MS/MS	[35]
Pear pomace (*Pyrus communis* L., var. Rocha) flours	Tocopherols	α-tocopherol (3.418–5.825) β-tocopherol (0.537–0.836) γ-tocopherol (0.378–0.606) δ-tocopherol (0.03–0.126)	UHPLC–fluorescence	[2]
Pineapple residue (*Ananas comosus* L. Merril)	Carotenoids	β-carotene (1.65–16.09)	UV–Vis spectroscopy	[13]
Tomato residue	Carotenoids	Lycopene (98.4–143.1)	HPLC–DAD	[14]
Pumpkin (*Cucurbita Maxima*) peel, flesh, and seeds	Carotenoids	β-carotene (9.9–61.8)	HPLC–DAD	[65]
Kinnow (*Citrus reticulata*) peel	Carotenoids	Lutein (29.7)	HPLC–DAD	[111]
Soybean residue (okara)	Fatty acids	Oleic acid (19.6–24.2%) Linoleic acid (49.4–55.1%)	GC–FID	[19]
Sour cherry (*Cerasus Vulgaris Miller*) kernel oil	Fatty acids	Linoleic acid (42.34%) Oleic acid (35.45%) Palmitic acid (6.54%) α-eleostearic acid (9.34%)	GC–FID	[91]
Eggplant fruit (*Solanum melongena* L.) pulp	Fatty acids	Palmitic acid (44.8%) Stearic acid (24.4%)	GC–FID	[6]
Cocoa (*Theobroma cacao*) waste flours	Fatty acids	Palmitic acid (26.8–34.3%) Stearic acid (3.1–15.8%) Oleic acid (7.6–36.8%) Linoleic acid (15.7–48.9%) Linolenic acid (2.2–3.6%)	GC–FID	[61]

**Table 4:** Methods and bioactive properties of agri-food bio-residues.

Residue	Methods Applied	Bioactive Properties	Ref.
Pear pomace (*Pyrus communis* L.) flour	ABTS (2.3–3.0 mmol TE·100 g^−1^) ORAC (5.3–6.1 mmol TE·100 g^−1^) AChEi (15.10–23.43%) BChEi (9.61–16.04%)	Antioxidant Neuroprotective	[2]
Plum (*Prunus domestica* L.) seed	DPPH (10–36 mg·mL^−1^/0.9–1.9 mg·g^−1^) TBARS (1.3–5.0 mg·g^−1^) *E. coli* and *S. aureus* (MIC ≥ 20 mg·mL^−1^) Aβ aggregation inhibition (TEM) Aβ fibrils width reduction (30%)	Antioxidant Antibacterial Neuroprotective	[3]
Brazilian berry (*Eugenia brasiliensis* Lam.)	TBARS (0.90–1.34 µg·mL^−1^) Gram-positive bacteria (MIC = 0.078–2.5 mg·mL^−1^) Gram-negative bacteria (MIC = 0.078–20 mg·mL^−1^) *A. fumigatus* and *A. brasiliensis* (MIC = 0.31–1.25 mg·mL^−1^) LPS-NO (EC_50_ = 98–400 µg·mL^−1^) SRB (GI_50_ = 14.7–186.0 µg·mL^−1^)	Antioxidant Antibacterial Antifungal Anticarcinogenic Anti-inflammatory	[5]
Eggplant fruit (*Solanun melongena* L.)	TBARS (EC_50_ = 135–4300 µg·mL^−1^) Gram-positive and Gram-negative bacteria (MIC = 2–8 mg·mL^−1^; MBC = 4–8 mg·mL^−1^) *P. ochrochloron* (MIC = 1 mg·mL^−1^; MFC = 1 mg·mL^−1^) SRB (GI_50_ = 280–340 µg·mL^−1^)	Antioxidant Antibacterial Antifungal Anticarcinogenic	[6]
Chestnut (*Castanea sativa* Mill.) burs, shells, and leaves	DPPH (EC_50_ = 0.07–0.90 mg·mL^−1^) TBARS (EC_50_ = 0.002–2.0 mg·mL^−1^) FRAP (EC_50_ = 0.07–1.34 mg·mL^−1^) Gram-positive and Gram-negative bacteria (MIC ≤ 10 mg·mL^−1^; MBC = 5 mg·mL^−1^) *A. fumigatus* and *A. brasiliensis* (MIC = 10 mg·mL^−1^)	Antioxidant Antibacterial Antifungal	[10]
Quince (*Cydonia oblonga* Mill.)	DPPH (72% inhibition) ABTS (380 µmol TE·100 g^−1^)	Antioxidant	[122]
Pomegranate (*Punica granatum*) peel	ABTS (15–30 mM TE) FRAP (45–100 mmol iron eq.·L^−1^) Gram-positive and Gram-negative bacteria (agar diffusion method < 4 mm)	Antioxidant Antibacterial	[98]
Alperujo *Arbequina* and *Coratina*	ABTS (0.607–1.224 mg TE·g^−1^) ORAC (180–318 mg TE·g^−1^) Gram-positive and Gram-negative (MIC = 0.6–1.3 mg·mL^−1^)	Antioxidant Antibacterial	[36]
Saw palmetto (*Serenoa repens*)	ABTS (39–140 mg TE·g^−1^) DPPH (0.031–0.088 mg TE·g^−1^) Gram-positive and Gram-negative bacteria (MBC = 12.5–100 mg·mL^−1^)	Antioxidant Antibacterial	[123]
Acerola cherry (*Malpighia emarginata*)	DPPH (3.5 g TE·100 g^−1^) Gram-positive and Gram-negative bacteria (disk diffusion method = 7.9–8.2 mm)	Antioxidant Antibacterial	[105]
Oregano (*Origanum vulgare*)	DPPH–ABTS–FRAP (300–450 mg GAE·g^−1^) Gram-positive and Gram-negative bacteria (20% inhibition)	Antioxidant Antibacterial	[102]
Rosemary (*Rosmarinus officinalis*)	DPPH–ABTS–FRAP (250–300 mg GAE·g^−1^) Gram-positive and Gram-negative bacteria (100% inhibition)		
Sage (*Salvia fruticosa*)	DPPH–ABTS–FRAP (300–350 mg GAE·g^−1^) Gram-positive and Gram-negative bacteria (100% inhibition)		
Lemon balm (*Melissa officinalis*)	DPPH–ABTS–FRAP (500–700 mg GAE·g^−1^) Gram-positive and Gram-negative bacteria (20% inhibition)		
Spearmint (*Mentha spicata*)	DPPH–ABTS–FRAP (350–500 mg GAE·g^−1^) Gram-positive and Gram-negative bacteria (100% inhibition)		

## Abbreviations

ABTS, 2,2′-azino-bis(3-ethylbenzothiazoline-6-sulfonic acid); AChE, acetylcholinesterase; ACN, acetonitrile; Aβ_42_, amyloid-beta peptide; BChE, butyrylcholinesterase; bioSUPRAS, supramolecular biosolvents; BSA, bovine serum albumin; CAE, caffeic acid equivalents; CE, catechin equivalents; CSLE, conventional solid-liquid extraction; CUPRAC, cupric reducing antioxidant capacity; DAD, diode-array detector; DESs, deep eutectic solvents; DPPH, 2,2-diphenyl-1-picrylhydrazyl; EC_50_, half maximal effective concentration; FAMES, fatty acid methyl esters; FRAP, ferric reducing antioxidant power; GAE, gallic acid equivalents; GC, gas chromatography; GI_50_, concentration required to inhibit 50% of the cell growth; HF, formic acid; HPLC, high-performance liquid chromatography; IC_50_, concentration required to inhibit 50%; LC, liquid chromatography; LPS, lipopolysaccharide; *m*/*z*, mass-to-charge ratio; MAE, microwave-assisted extraction; MBC, minimum bactericidal concentration; MDA, malondialdehyde; MFC, minimum fungicidal concentration; MIC, minimum inhibitory concentration; MS, mass spectrometry; MS/MS, tandem mass spectrometry; MSPD, matrix solid-phase dispersion; MTT, 3-(4,5-dimethylthiazol-2-yl)-2,5-diphenyltetrazolium bromide; MUFA, monounsaturated fatty acids; NADESs, natural deep eutectic solvents; ORAC, oxygen radical absorbance capacity; PEF, pulsed electric fields; PFE, pressurized fluid extraction; PLE, pressurized liquid extraction; PUFA, polyunsaturated fatty acids; Q, quadrupole analyzer; QE, quercetin equivalents; QqQ, triple quadrupole analyzer; QTOF, quadrupole time-of-flight analyzer; RE, rutin equivalents; RP, reverse phase; SDGs, Sustainable Development Goals; SFA, saturated fatty acids; SFE, supercritical fluid extraction; SRB, sulforhodamine B; TAC, total anthocyanin content; TAE, tannic acid equivalents; TAs, tropane alkaloids; TBA, thiobarbituric acid; TBARS, thiobarbituric acid reactive substances; TE, trolox equivalents; TEM, transmission electron microscopy; TF, total flavonol; TFC, total flavonoid content; THA, total hydroxycinnamic acid; TOF, time-of-flight analyzer; TPC, total polyphenol content; UAE, ultrasound-assisted extraction; UHPLC, ultra-high-performance liquid chromatography.
